# Targets and strategies to design soybean seed composition traits

**DOI:** 10.1002/tpg2.70115

**Published:** 2025-09-26

**Authors:** Ritesh Kumar, Steven Mulkey, Rahul Mahadev Shelake, Rachel Combs‐Giroir, Thiya Mukherjee, Doug K. Allen, Tom Elmo Clemente, Minviluz G. Stacey, Aaron J. Lorenz, Robert M. Stupar

**Affiliations:** ^1^ Department of Agronomy and Plant Genetics University of Minnesota Saint Paul Minnesota USA; ^2^ Ball Horticultural West Chicago Illinois USA; ^3^ Division of Applied Life Science (BK21 Four Program), Plant Molecular Biology and Biotechnology Research Center Gyeongsang National University Jinju Republic of Korea; ^4^ Donald Danforth Plant Science Center St. Louis Missouri USA; ^5^ Department of Natural Sciences Texas A&M University‐San Antonio San Antonio Texas USA; ^6^ Department of Agronomy & Horticulture University of Nebraska‐Lincoln Lincoln Nebraska USA; ^7^ Division of Plant Sciences University of Missouri Columbia Missouri USA

## Abstract

Breeders and geneticists have put great effort into enhancing soybean seed composition and developing elite varieties with desired traits. However, diverse end‐uses and changing consumer preferences present new challenges and opportunities to develop desired compositional profiles for the market. Recent advances in genetics and novel technologies have allowed researchers to characterize genes impacting seed composition and provide a means for addressing these shifting consumer needs. Some of the desired compositional traits for soybean [*Glycine max* (L.) Merr.] include increased levels of protein, oil, sucrose, sulfur‐containing amino acids, and omega‐3 fatty acids, and reduced allergens, raffinose family oligosaccharides, or saponins. This comprehensive review considers the current status of key components of soybean seed composition and forecasts opportunities based on candidate genes/pathways that may be targeted to add seed value. The review addresses a holistic view on interactions between the genome, transcriptome, proteome, and epigenome, along with metabolic flux analysis to gain insights into biological underpinnings governing seed composition.

## Core Ideas

- The demand for soybean seed composition traits varies according to the end user's requirements.
- Diverse end‐uses and changing consumer preferences pose new challenges and opportunities for breeders to alter current compositional profiles and develop new varieties with tailored traits.
- Multiple omics approaches and genome editing tools may facilitate progress in soybean seed composition work.
- Metabolic flux analysis provides insights on carbon partitioning that contribute to our understanding of omic investigations and can guide engineering efforts.

## Plain Language Summary

Soybean seeds are a valuable source of plant‐based protein, oil, and other components used in food, animal feed, biofuels, and other industrial applications. Modifying the compositional profile of soybean seeds to suit these diverse uses is desirable; however, the relative abundance of key seed constituents is difficult to change through traditional breeding alone. A deeper understanding of the genes involved in these metabolic pathways may enable the use of biotechnology to overcome current limitations. This paper reviews the current knowledge of the genes and pathways that regulate soybean seed composition traits and highlights key targets for improvement.

**Abbreviations**
ACCase: acetyl‐CoA carboxylase
ACP: acyl carrier protein
ASN: asparagine
BBI: Bowman–Birk inhibitor
CD: central dogma
CGS: cystathionine gamma‐synthase
CHO: carbohydrates
CRISPR: clustered regularly interspaced short palindromic repeats
CYS: cysteine
DAG: diacylglycerol
DDMP: 2,3‐dihydro‐2,5‐dihydroxy‐6‐methyl‐4H‐pyran‐4‐one
DGAT: diacylglycerol acyltransferase
DM: dry mass
EMS: ethyl methane sulfonate
FA: fatty acid
GLY: glycine
HOLL: high oleic/low linolenic acid
Ig: immunoglobulin
KTI: Kunitz trypsin inhibitor
LMW: low‐molecular‐weight
MET: methionine
MGL: methionine γ‐lyase
QTL: quantitative trait locus
RFOs: raffinose family oligosaccharides
RS2: raffinose synthase
SAM: S‐adenosylmethionine
SMM: S‐methylmethionine
SSC: soybean seed composition
STS: stachyose synthase
TAG: triacylglycerol
TI: trypsin inhibitor

## 1 INTRODUCTION

**FIGURE 1:**
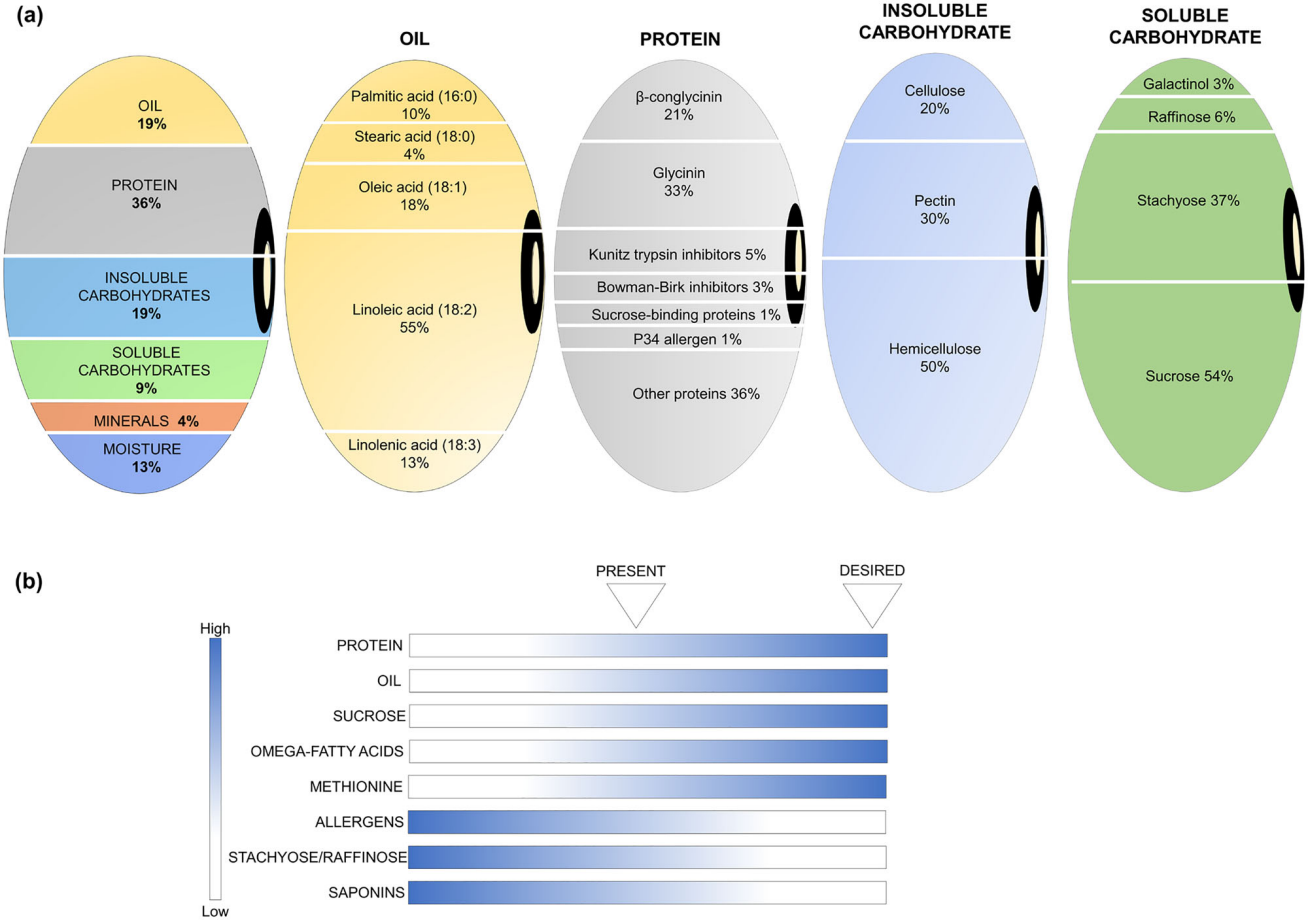
Approximate seed composition profile of present‐day commodity soybean and potential desired alterations to fit specific market demands. (a) Soybean seed can be partitioned on the percentage basis of each component, such as oil, protein, and soluble and insoluble carbohydrates. Furthermore, each component is individually elaborated according to its sub‐constituents (e.g., fatty acids composing the oils). (b) The present versus desired composition for meeting specific market demands for food, feed, and fuel. Blue represents higher abundance, while white represents lower abundance. Desired values vary depending on the end‐use product. Values in (a) are approximated based on previous publications (e.g., Chaudhary et al., 2015; Clemente & Cahoon, 2009; Saldivar et al., 2011) but can vary widely depending on genetics and environment.

The economic success of soybean [*Glycine max* (L.) Merr.] as a legume commodity crop is owed to its unique seed composition, with value derived from both oil and protein (Figure 1), and the ability to fix atmospheric nitrogen to ammonia through symbiosis with rhizobia. Depending on the genotype and environment in which it is grown, harvested soybean seed is comprised of approximately 36% protein and 19% oil, with additional reserves in the form of soluble and non‐soluble carbohydrates (CHOs), metabolites, and minerals (Medic et al., 2014; P. Singh & Krishnaswamy, 2022) (Figure 1a). Altering the type and content of these constituents holds potential to expand the soybean market and is thus an active area of research (Chaudhary et al., 2015; Clemente & Cahoon, 2009; Saldivar et al., 2011) that has been an ongoing priority for the soybean community (Boerma et al., 2011; Stupar et al., 2024).

Soybeans are processed using either solvent extraction or mechanical pressing to separate oil from the remaining fractions. The oil fraction is primarily used in food and industrial applications, while the remaining meal serves as a high‐quality protein source in feed rations for poultry, swine, cattle, and aquaculture (Sørensen et al., 2009). Soybeans are deficient in the sulfur‐containing amino acids methionine (MET) and cysteine (CYS). Hence, feed formulations often require supplementation of synthetic sulfur‐containing or other amino acids (Krishnan, 2015). The CHOs content of soybeans, including sucrose, raffinose, and stachyose, also impacts feed quality (Wilson, 2004). Enhanced sucrose content is desirable as a metabolizable energy source in animal feed; however, raffinose and stachyose cause indigestibility and flatulence and negatively affect feed conversion efficiency (Hagely et al., 2013), as do trypsin inhibitors (TIs), isoflavones, and saponins (W. Chen et al., 2011).

A similar set of challenges exists for soy protein destined for human consumption. It is estimated that approximately 0.3% of people around the globe are allergic or sensitive to soy protein (Messina et al., 2022). Soybean contains 16 immunoglobulin E (IgE) reactive proteins, which are allergenic (Ballmer‐Weber & Vieths, 2008). The major allergens in soybean include Kunitz trypsin inhibitors (KTIs), thiol‐protease P34/Gly m Bd 30k, storage proteins like the α subunit of β‐conglycinin, the acidic chain of the glycinin gy1 subunit, and basic chain of the glycinin gy2 subunit (summarized in Mulalapele & Xi [2021]). Additionally, soy protein contains enzymes such as lipoxygenase, which can create undesirable “beany” flavors in food products. Furthermore, saponins and isoflavones may contribute to bitter or soapy flavors (Aldin et al., 2006).

A challenge to improving soybean seed composition (SSC) is that modifying individual constituents often affects other reserves. Increasing protein results in reduced oil production (Patil et al., 2017) and SSC traits negatively correlate with harvestable yield (Bandillo et al., 2015; Chung et al., 2003; M. Kim et al., 2016; Patil et al., 2017). The SSC variation within the soybean germplasm is driven by factors including genetic, post‐transcriptional, epigenetic, and environmental (M. Kim et al., 2016; Nichols et al., 2007; Patil et al., 2017, 2018; Sebolt et al., 2000).

Soybean breeding programs have significantly improved germplasm by favorably altering fatty acids (FAs) composition in soybean oil (Amjad Khan et al., 2017; K. Bilyeu et al., 2005). Oleic acid is resistant to oxidation relative to polyunsaturates, extending the shelf life that is desirable for cooking. High oleic soybeans have been developed by mutating fatty acid desaturase (*FAD2* & *FAD3*) genes, resulting in less linolenic acid and more oleic acid (Buhr et al., 2002; Lee et al., 2012; Pham et al., 2010, 2011). These mutations can be combined with other mutations to improve seed quality (K. Bilyeu et al., 2018). Genetic improvements reduce the requirement for chemical hydrogenation that produces unhealthy trans fats. An in‐depth study of the oil biosynthesis pathway using multiple omics approaches, and newer crop varieties with value‐added traits, would provide a better understanding of the molecular basis of soybean oil improvement. Although breeding programs have addressed some of the issues mentioned earlier, the preferred outcomes are impeded by a lack of genetic variability for the desired traits and limited knowledge about genetic factors influencing the desired phenotype (Pramanik et al., 2021). Understanding the genetic underpinnings governing SSC is crucial to the development of soybean varieties with desired seed constituents. Genetic strategies and approaches that can be used to complement soybean breeding programs to address future SSC are discussed below.

Advances in the ability to edit endogenous and/or add transgenic alleles, omic capacities, and flux analyses have primed soybean for significant improvements in composition (Figure 1b). Genetic resources available for soybean include multiple reference genome assemblies (M. J. C. Espina et al., 2024; Schmutz et al., 2010; Tian et al., 2025; Valliyodan et al., 2019) and resequencing datasets (J. S. C. Chu et al., 2021; J. Y. Liu, Du, et al., 2020; Torkamaneh et al., 2021; Zhou et al., 2015), genome‐wide omics (messenger RNA [mRNA]/protein/metabolite expression) data (Fang et al., 2017; L. Li et al., 2015; J. Y. Liu, Li, et al., 2020; Lu et al., 2021; Min, Gupta, et al., 2020; Min, Park, et al., 2020; Qi et al., 2018; Schmidt et al., 2011; Shamimuzzaman & Vodkin, 2018; B. Song et al., 2016; Yang et al., 2019), mutant populations (Bolon et al., 2011; M. J. Espina et al., 2018; M. Zhang et al., 2022), known seed composition quantitative trait loci (QTLs) (e.g., Fliege et al., 2022; Goettel et al., 2022; Phansak et al., 2016; Zheng et al., 2025), genetic/genome engineering technologies (Clemente et al., 2000; Karlson et al., 2021; C. Li, Nguyen, et al., 2019; J. Liu et al., 2019; Lowder et al., 2015; Pan et al., 2022, 2021; Z. Zhang et al., 1999) and studies of metabolic flux (Allen et al., 2009; Allen & Young, 2013; Iyer et al., 2008; Morley et al., 2023; Sriram et al., 2004). These resources provide opportunities to expand soybean end‐uses and markets.

## 2 MODIFYING SOYBEAN MEAL COMPOSITION

### 2.1 Protein

#### 2.1.1 Protein components

**FIGURE 2:**
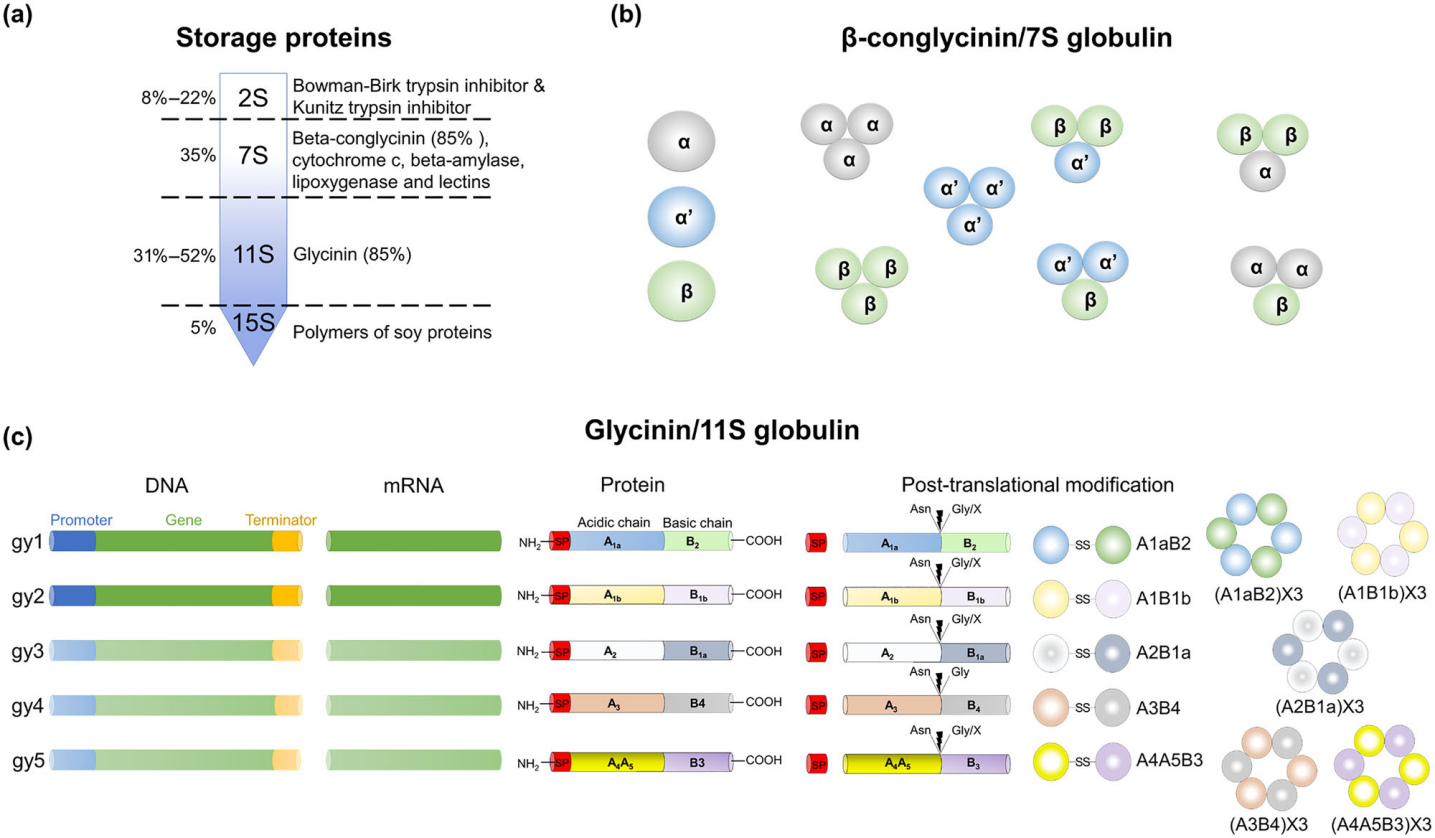
Major soybean seed storage proteins. (a) Soybean seed is partitioned based on sedimentation rate. β‐Conglycinin and glycinin are the two dominant seed storage proteins. (b) Subunits of β‐conglycinin and assemblies after post‐translational modification. Three subunits make seven different combinations. (c) Major proteins belong to the glycinin protein family, ranging from glycinin 1 (gy1) through glycinin 5 (gy5). Each protein comprises unique acidic and basic peptide chains that cleave at the junction of asparagine (ASN), glycine (GLY), or another conserved amino acid. The acidic peptide chain on the N‐terminal and the basic peptide in the C‐terminal are joined together by the double sulfur bond to make a dimer. Later, post‐translational modifications result in hexamer formation.

Soybean seed protein reserves are partitioned into four categories, based on molecular weight as determined by sedimentation rate, representing varying percentages in the final protein content as follows: 2S (8%–22%), 7S (35%), 11S (31%–52%), and 15S (5%) (L'Hocine & Boye, 2007). Each fraction represents a family of proteins with different characteristics and biological functions (Figure 2a). The 2S fraction contains the Bowman–Birk inhibitors (BBI) and KTI family members, which are anti‐nutritional components of soybean meal as feed for swine, foul, and potentially fish. The 7S fraction is primarily composed of β‐conglycinin (∼85%), cytochrome c, β‐amylase, lipoxygenases, and lectins. The 11S fraction is composed mainly of glycinin (∼85%), and the 15S fraction is not yet fully characterized (L'Hocine & Boye, 2007). Collectively, the SSC is dominated by two major storage protein families, β‐conglycinin and glycinin (Murphy, 2008), which have been previously reported to constitute approximately 60% of the total seed protein (Koshiyama, 1968; Schmidt et al., 2011; Thanh & Shibasaki, 1976).

**Core Ideas**

- The demand for soybean seed composition traits varies according to the end user's requirements.
- Diverse end‐uses and changing consumer preferences pose new challenges and opportunities for breeders to alter current compositional profiles and develop new varieties with tailored traits.
- Multiple omics approaches and genome editing tools may facilitate progress in soybean seed composition work.
- Metabolic flux analysis provides insights on carbon partitioning that contribute to our understanding of omic investigations and can guide engineering efforts.

β‐Conglycinin is comprised of three subunits named α, α′, and β. Historically 15 genes were reported to encode the β‐conglycinin family, designated as *CG‐1* to *CG‐15*, with limited information on the link between the gene model and translational product. C. Li and Zhang (2011) exploited the protein sequences of CG‐1 to CG‐4 to connect gene models to translational products. Nine homologous sequences distributed across soybean chromosomes 2, 10, and 20 revealed the gene models composing the β‐conglycinin family using the original soybean genome assembly (Wm82.a1.v1.1; Schmutz et al., 2010). *Glyma10g39150* encodes the α′‐subunit, whereas *Glyma20g28650* and *Glyma20g28660* encode the α‐subunit, and *Glyma20g28460* and *Glyma20g28640* encode the β‐subunit (C. Li & Zhang, 2011). C. Li and Zhang (2011) also reported related storage proteins, including the β‐conglycinin family members *Cgy1* (gene model *Glyma10g39170*) and the sucrose binding proteins (encoded by gene models *Glyma02g16440* and *Glyma10g03390*), and the pseudogene *Glyma10g39160*. The version 4 Williams 82 genome release listed in Phytozome (https://phytozome‐next.jgi.doe.gov/) (Wm82.a4.v1; Valliyodan et al., 2019) has six functional gene models annotated for the three β‐conglycinin subunits, *Glyma.10g246300* and *Glyma.10g246500* (α′‐subunit), *Glyma.20g148300* and *Glyma.20g148400* (α‐subunit), and *Glyma.20g146200* and *Glyma.20g148200* (β‐subunit). Post‐translational modifications allow for the trimerization of these subunits found in seven different combinations (Figure 2b).

The ratio of glycinin subunits, initially designated gy1–gy5 by Nielsen et al. (1989), exhibited variation among soybean genotypes. The molecular evolution and phylogenetic relationship of the glycinin family is categorized into three subfamilies (C. Li & Zhang, 2011), with gy1, gy2, and gy3 representing one, gy6 and gy7 the second, and the third consisting of gy4 and gy5 (Beilinson et al., 2002; C. Li & Zhang, 2011).

Transcript levels of gene models annotated for gy6 and gy7 are very low (Beilinson et al., 2002), effectively reducing the functional glycinin members to five. However, the number of functional glycinin gene models varies between genotypes (Žilić et al., 2011). Each glycinin subunit contains unique acidic and basic polypeptide domains at the N‐terminal and C‐terminal ends, respectively, with a cleavage point that typically resides between asparagine (ASN) and glycine (GLY) residues. The acidic and basic domains of a subunit are dimerized via two sulfur bonds and post‐translationally modified, resulting in hexamers (Figure 2c) (C. Li & Zhang, 2011). The post‐translational modifications of glycinin and β‐conglycinin subunits may affect their interactions with one another; however, the details and functional relevance of these interactions remain unknown.

**FIGURE 3:**
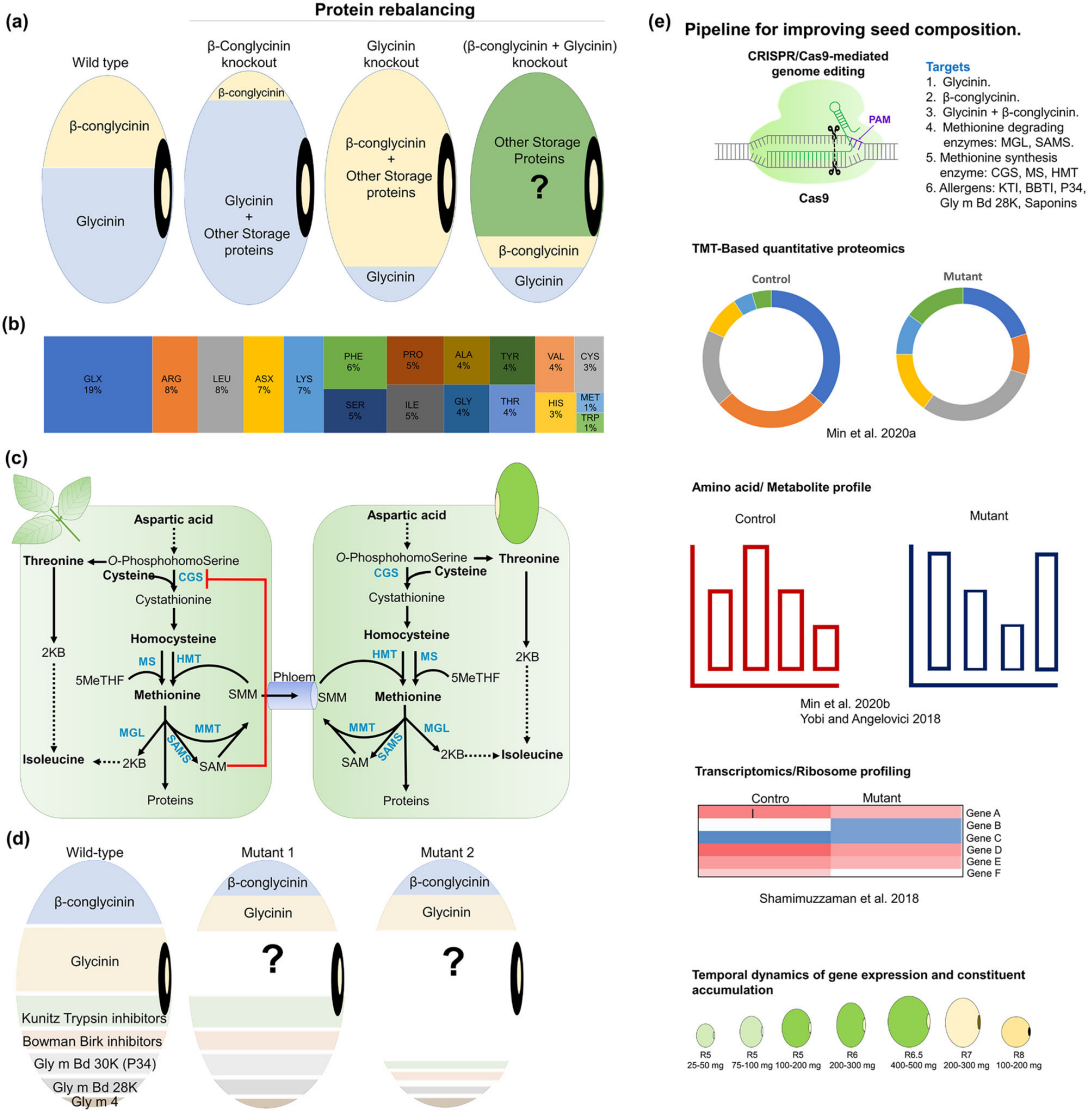
Soybean protein rebalancing and pathway to new traits. (a) Concept of protein rebalancing with the example of β‐conglycinin and glycinin storage proteins. The wild type represents the typical ratio of β‐conglycinin and glycinin proteins in soybean seeds. β‐Conglycinin knockouts may result in higher glycinin and other storage proteins. Glycinin knockouts may show compensation by β‐conglycinin and other storage proteins. These processes are known as protein rebalancing. (b) Average amino acid composition of mature soybean seed protein. GLX stands for pool of (GLU and GLN) and ASX represents the pool of (ASN and ASP). Alanine: ALA, Arginine: ARG, Asparagine: ASN, Aspartic acid: ASP, Cysteine: CYS, Glutamic acid: GLU, Glutamine: GLN, Glycine: GLY, Histidine: HIS, Isoleucine: ILE, Leucine: LEU, Lysine: LYS, Methionine: MET, Phenylalanine: PHE, Proline: PRO, Serine: SER, Threonine: THR, Tryptophan: TRP, Tyrosine: TYR, Valine: VAL. (c) Biosynthetic pathway of sulfur‐rich methionine (MET). Initially, MET is synthesized in leaves and transported to seeds in the form of S‐methylmethionine (SMM). The red loop represents the negative feedback inhibition of cystathionine gamma‐synthase (CGS) by S‐adenosylmethionine (SAM). CGS is thought to be a rate‐limiting enzyme in MET biosynthesis. (d) Main soybean allergens in the wild‐type seed and subsequent alteration in allergen abundance in hypothetical mutant 1 and mutant 2. In hypothetical mutant 1, mutating β‐conglycinin and glycinin gene family members may have an additive effect on seed proteome and rebalancing. In hypothetical mutant 2, multiple storage proteins and allergens can be mutated to see effects on the seed proteome. (e) Pipeline for improving seed composition. The genes responsible for undesired traits could be knocked out using clustered regularly interspaced short palindromic repeats (CRISPR)/Cas9 with single or multiple targets. The mutants could then be examined using a multi‐omics approach: proteomics (posttranslational modifications like phosphorylation and ubiquitination) (Min, Gupta, et al., 2020), metabolomics (Min, Park, et al., 2020; Yobi & Angelovici, 2018), and/or transcriptomics/ribosome profiling (Shamimuzzaman & Vodkin, 2018). To access the key regulators in protein rebalancing, the multi‐omics study can be carried out from the beginning of seed development to the end. HMT, homocysteine methyltransferase; MGL, methionine γ‐lyase; MMT, Met methyltransferase; MS, methionine synthase; PAM, protospacer adjacent motif; SAMS, S‐adenosylmethionine synthase.

A phenomenon known as proteome rebalancing (Herman, 2014) is triggered when a genetic mutation and/or deliberate down‐regulation of a major seed storage protein gene(s) is imposed during seed development. Other storage proteins increase production to compensate such that the total protein amount remains largely unchanged. Depending on which protein types get upregulated, rebalancing can alter the protein nutritional and functional qualities of the seed. This phenomenon is well‐documented in the case of β‐conglycinin and glycinin (W. S. Kim, Jez, et al., 2014; Wei et al., 2020) as well as KTI and BBI (Gillman et al., 2015) (Figure 3a). Efforts have been made to generate storage protein mutants using targeted mutagenesis and RNA interference (RNAi) (Gillman et al., 2015; Herman, 2014; W. S. Kim, Jez, et al., 2014; Schmidt et al., 2011; B. Song et al., 2017; Wei et al., 2020), during which protein rebalancing was observed. The mechanism(s) by which proteome rebalancing occurs are poorly understood, and their elucidation holds potential for informing the development of soybean genotypes with varying protein functionalities for specific end‐uses. The major storage proteins in soybean are summarized in Table S1.

#### 2.1.2 Genetic strategies to trigger proteome rebalancing

Previous investigations into the mechanism underlying proteome rebalancing have examined the relationship between β‐conglycinin and glycinin. C. Li, Nguyen et al. (2019) used genome editing reagents to generate null mutations of the highly homologous β‐conglycinin subunits. These biologicals, along with a set of edits that vary for null mutations in other soybean seed protein reserve gene families, would be a valuable resource for enabling multiple‐omics approaches that assess dose‐dependent effects and their impact on proteome rebalancing.

Recently, edited soybean lines carrying null mutations in multiple members of both β‐conglycinin (7S) and glycinin (11S) were characterized by Bai et al. (2022). The outcome revealed no changes in total protein and oil relative to controls, consistent with proteome rebalancing. Assessment of the functional properties of the protein reserves in the edited lines showed emulsions prepared from 11S‐null mutants had higher stability, while stability was reduced in emulsions from 7S‐null. The 7S‐null displayed increased gelling hardness, springiness, gumminess, and chewiness compared to control proteins (Bai et al., 2022). In contrast, the 11S‐null mutants exhibited protein with a significant decrease in these parameters.

**FIGURE 4:**
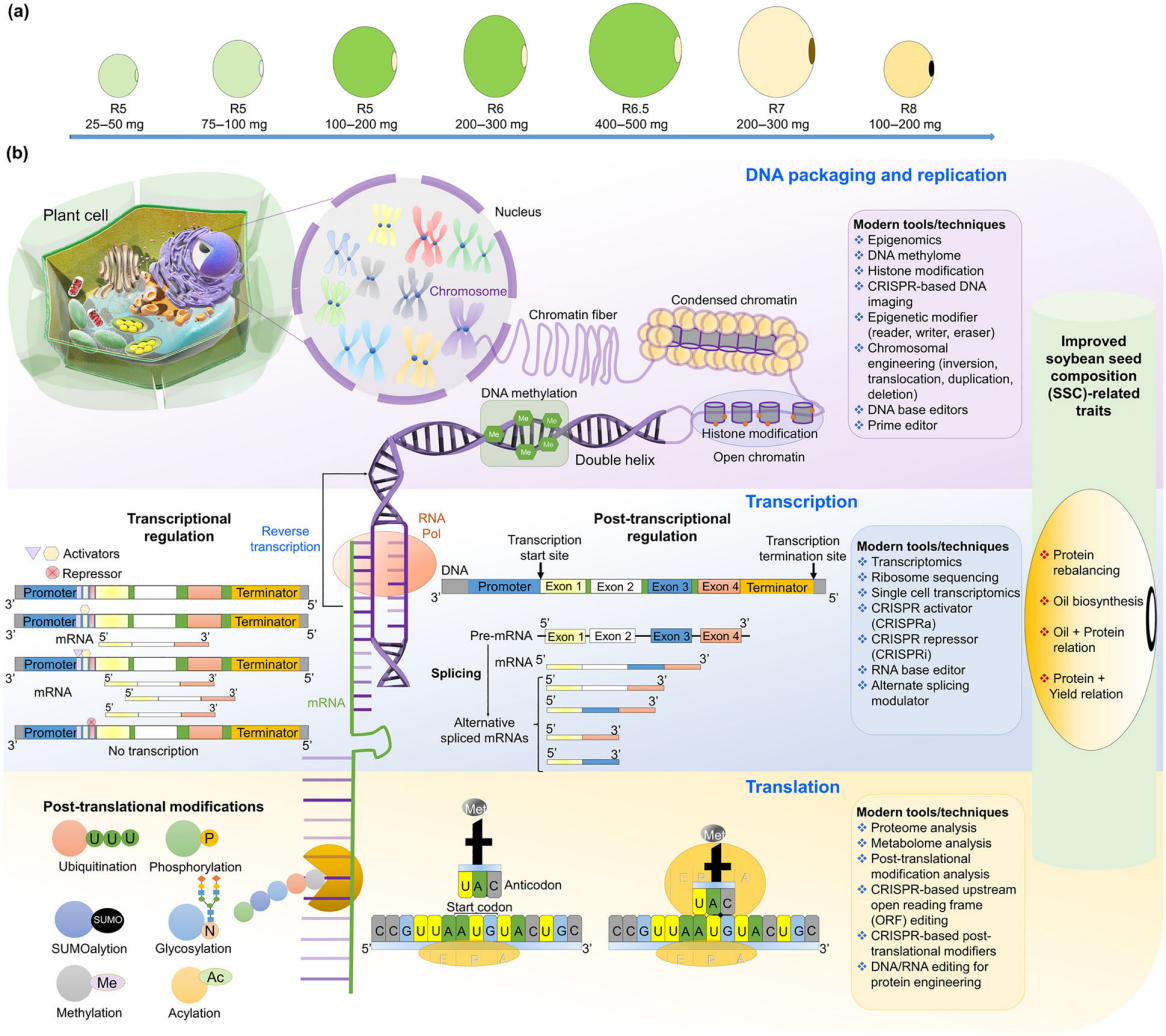
Modification of central dogma (CD) processes governing the desired composition in soybean seeds. (a) The soybean seed development stages range from R5 to R8 based upon fresh weight (mg) from left to right; (b) outline of CD processes and modern tools for CD engineering leading to altered soybean seed composition (SSC) is summarized. The CD processes transmitting genetic information from DNA to RNA to protein modified from Pramanik et al. (2021). DNA packaging is a critical factor in regulating genome stability and integrity. DNA packaging can be observed as chromosomes, which are packaged as chromatin fibers, to nucleosomes, down to the double‐stranded DNA molecule. Alterations in the chromatin, such as DNA methylation (Me) and histone modifications, are essential factors in controlling epigenetic memory. Specific activators and repressors regulate the transcriptional state of the DNA, resulting in mRNA. Post‐transcriptional mRNA processing involves alternative splicing to make the mRNA stable and available for translation. After translation, proteins can be modified by several biochemical processes like phosphorylation, glycosylation, ubiquitination, methylation, acylation, and polyamination (collectively known as post‐translational modifications), which adds one more dynamic and complex layer to biological processes. On the right, modern tools and techniques are summarized to explore different stages of CD processes responsible for SSC that may help in the design of needs‐based SSC alteration. The 3D model of the plant cell was obtained from Microsoft PowerPoint Stock Models.

Proteomics and transcriptomics datasets collected from immature seeds at early to mid‐reproductive stages have provided a glimpse into the genetic controls influencing protein deposition during seed development (Jones & Vodkin, 2013; Plumblee & Harrelson, 2022) and resulted in comparisons with other oilseeds (Agrawal et al., 2008). The development of soybean lines carrying 11S and 7S null mutations, and other gene models that are predicted to impact seed protein content, coupled with deep‐omics studies across development (Figure 4a), may provide insight into the control points of seed protein content/functionality.

### 2.2 Sulfur‐containing amino acids

#### 2.2.1 Methionine Metabolism

Free Met (fMet) is utilized in plant cells not only for protein biosynthesis but also as a precursor of important metabolites such as ethylene, phytosiderophores, and polyamines (Roje, 2006). The first committed step in fMet biosynthesis is catalyzed by cystathionine γ‐synthase (CGS), which catalyzes the addition of sulfur moiety from CYS to *O*‐phosphohomoserine (Amir, 2010). During seed filling, fMet produced in leaves is likely transported to seeds in the form of S‐methylmethionine (SMM), which is generated through the SMM cycle (Cohen et al., 2017) (Figure 3b). The production of fMet is tightly regulated by the feedback inhibition of CGS by S‐adenosylmethionine (SAM), a metabolite derived from fMet catabolism. Work in arabidopsis [*Arabidopsis thaliana* (L.) Heynh.] found that point mutations or deletions in the AtCGS MTO1 region (amino acids 77–87) resulted in feedback‐insensitive CGS and significantly increased fMet levels in mutant tissues (Hacham et al., 2006; Inaba et al., 1994; Ominato et al., 2002). In addition to SAM, fMet is also catabolized by methionine γ‐lyase (MGL) into methanethiol, ammonia, and 2‐ketobutyric acid, an intermediate in Isoleucine (ILE) biosynthesis (Joshi & Jander, 2009; Sato & Nozaki, 2009).

#### 2.2.2 Enhancing methionine levels in soybean seed storage proteins

The total amino acid content in seeds represents the combined levels of protein‐bound and free amino acids, with the latter contributing only 0.3%–0.8% of the seed nitrogen content (M. Takahashi et al., 2003). Therefore, the nutritional quality of seed proteins is mainly determined by the amino acid profile of seed storage proteins. Soybean is a superior protein source relative to that of other major commodities, such as distiller's dried grains with soluble derived from maize (*Zea mays* L.). However, as in many seeds, soybeans are deficient in Met (J. Chen et al., 2021), as Met is approximately 1% of total protein‐bound amino acids in most soybean genotypes (Figure 3c). Met and CYS are sulfur‐containing amino acids, and Met is an essential amino acid in human and animal diets (P. Singh & Krishnaswamy, 2022). Thus, its low abundance in soybeans negatively impacts the nutritional value of soy meal. Traditional breeding approaches identified several QTLs associated with soybean seed MET and CYS concentrations, but so far, these have not resulted in the release of commercial cultivars with increased sulfur amino acid content (Malle et al., 2020; Panthee et al., 2006; Singer et al., 2022).

In addition to traditional breeding, various transgenic approaches to increase protein‐bound Met in soybean have been tested by seed‐specific overexpression of heterologous Met‐rich storage proteins (Dinkins et al., 2001; Guo et al., 2020; W. S. Kim & Krishnan, 2004, 2019; Krishnan & Jez, 2018) with limited accumulation of heterologous seed proteins (W. S. Kim, Jez, et al., 2014). Interestingly, suppression of endogenous β‐conglycinin coupled with the seed‐specific expression of Met‐rich 11‐kDa δ‐zein and growth of transgenic plants on sulfur‐rich medium significantly increased the accumulation of δ‐zein levels by threefold to 16‐fold (W. S. Kim, Jez, et al., 2014). These results suggest that under normal growing conditions, the amount of fMet is limited for optimal translation of Met‐rich proteins in developing soybean seeds and this bottleneck was alleviated by sulfate supplementation. Likewise, overexpression of ATP sulfurylase, which impacts sulfur metabolism in plants, increased protein‐bound sulfur amino acid content: CYS by 37%–52% and Met by 15%–19% (W. S. Kim et al., 2020). Increased availability of fMet by overexpression of an arabidopsis feedback‐insensitive CGS (AtD‐CGS) in soybean seeds also resulted in enhanced levels of total Met and proteins in seeds (S. Song, Hou, et al., 2013; Y. Yu et al., 2018). Similar findings were obtained when AtD‐CGS was over‐expressed in arabidopsis seeds (Cohen et al., 2014).

Clearly, future efforts to biofortify Met in soybean should incorporate ways to increase fMet levels in developing seeds. This can be achieved through genetic manipulation to increase its biosynthesis, as mentioned above, and/or decrease its catabolism as recently shown in a soybean harboring a transposon insertion in *Glyma.10g172700* that encodes MGL, an fMet catabolic enzyme (Teshima et al., 2020). Lastly, targeted genome editing should be considered in engineering Met metabolism in seeds. Gene knockouts using clustered regularly interspaced short palindromic repeats (CRISPR)/Cas9 are well established in soybean and can be utilized to inactivate genes that encode fMet catabolic enzymes (e.g., MGL, S‐adenosylmethionine synthase). Likewise, precision tools such as base editing or prime editing can be utilized to enhance Met codons in endogenous soybean seed storage proteins. Although the success of these methods is limited in soybean, an era of continuous improvement in genome editing tools may make it feasible in the future. Met biofortification of seeds is reviewed in more detail in previous publications (Amir et al., 2019; Hesse & Hoefgen, 2003; Krishnan, 2015; Krishnan & Jez, 2018).

### 2.3 Soy allergens

#### 2.3.1 Characterization of soy allergens

On average, three out of 1000 people are allergic to soy protein, with varying symptoms, mostly mild. The allergenicity of soybean proteins is due to specific immunoreactive structures, that is, IgE‐binding epitopes (Errahali et al., 2002; X. Sun et al., 2013). In soybean‐sensitive individuals, IgE antibodies bind to multiple different soybean allergens, with the dominant allergens being Gly m Bd 60K, P34/Gly m Bd 30K, and Gly m Bd 28K, where Gly m stands for *Glycine max*, and numbers represent the protein molecular weight in kilodaltons (Wu et al., 2012).

The Gly m Bd 60K allergenic proteins are primary storage proteins: β‐conglycinin and glycinin (T. Wang et al., 2014; see Table S1 for gene model names). β‐Conglycinin has also been named Gly m 5 using the allergen nomenclature. All three β‐conglycinin subunits (α, α′, and β) have been demonstrated to have IgE binding activity (Krishnan et al., 2009). Glycinin has been named Gly m 6 using the allergen nomenclature. All the acidic and basic subunits of glycinin have demonstrated IgE binding activity in soybean‐sensitive patients (Helm, Cockrell, Connaughton, Sampson, Bannon, Beilinson, Livingstone, et al., 2000). However, the acidic part of the G1 subunit (A_1a_B_2_) appears to have much higher levels of IgE binding, even compared to the highly sequence homologous G2 (Zeece et al., 2010). Studies have shown that IgE binding occurs primarily along one plane outside these acidic peptides (Helm, Cockrell, Connaughton, Sampson, Bannon, Beilinson, Nielsen, et al., 2000). Multiple epitopes for each of these acidic subunits have been identified, and it appears that much of the IgE binding activity is due to a six‐residue region located within sequence S219‐N233 (Beardslee et al., 2000; Zeece et al., 2010).

Gly m Bd 30K is also called the P34 or 34 kDa seed maturing protein. The P34 protein is the “immunodominant” allergen source found in soybeans. Of at least 15 allergenic soybean proteins, P34 is responsible for the allergic response in over 65% of soy‐sensitive people (Messina et al., 2022). P34 (*Glyma.08g116900*) belongs to the papain superfamily of CYS proteinase type of enzyme (Kalinski et al., 1990). Specifically, the ontology for the protein describes it as a CYS‐type endopeptidase or a probable thiol protease. The first 15 N‐terminal amino acid residues of P34 are homologous to the papain family's thiol proteinase, including the dust mite thiol proteinase Der p1 (L'Hocine & Boye, 2007).

Another major allergen, Gly m Bd 28K (*Glyma.08g116900*), is homologous to MP27–MP33, a member of the cupin protein superfamily. In the sera of soybean allergic patients, the glycan moiety of *Gly m* Bd 28 K binds to IgE antibodies at Asparagine 20 (Hiemori et al., 2000). Another identified allergen is KTI, with multiple genes encoding proteins in developing seeds. Most known soybean genotypes with low KTI content in seeds carry the “null” “ti” allele (aka. KTI3), such as PI 157440, an accession from South Korea (Krishnamurthy et al., 2013). The null allele of *Glyma.08g341500* from PI 157440 was introgressed into Williams 82 background to develop the genotype “Kunitz,” which was subsequently used to create a “triple null” soybean line when stacked with mutated forms of P34 and Agglutinin (Schmidt et al., 2015). This “triple null” genotype was recently reconstituted in northern latitude germplasm using both CRISPR editing and conventional backcrossing (J. Liu et al., 2025). Germplasm carrying the original “triple null” alleles has a significant reduction in TI activity (around 40%) (Gillman et al., 2015). It was revealed that the genotypes exhibiting the reduced TI activity have a frameshift mutation in the ti/*KTI3* gene model *Glyma.08g341500*. Gillman et al. (2015) identified another soybean genotype, PI 68679, with a nonfunctional mutation in the *KTI1* gene model *Glyma.01g095000*. The stacking of *KTI1* and *KTI3* alleles has a synergistic effect in controlling the TI abundance in soybean seeds (Gillman et al., 2015). Recently, Z. Wang et al. (2023) confirmed KTI family members *KTI1* and *KTI3* are indeed seed‐specific TI genes. They generated knockout mutants *kti1* and *kti3* in the soybean cultivar Williams 82 using CRISPR‐Cas9. The double mutant *kti1/kti3* displayed significantly reduced KTI content and TI activity relative to wild type. Previously, it was found that the reduction in KTI content was coincident with an increase in the BBI expression (Gillman et al., 2015). Eight different gene models have been annotated to encode for BBI proteins in Williams 82 soybean reference genome version Wm82.a4.v1 (Valliyodan et al., 2019). These include *Glyma.09g158900*, *Glyma.09g158500*, *Glyma.14g117700*, *Glyma.09g158600*, *Glyma.09g158700*, *Glyma.09g158800*, *Glyma.14g117600*, and *Glyma.16g208900*. Evaluation of 12,690 soybean accessions maintained by the USDA (Domagalski et al., 1992) showed that all wild soybean (*Glycine soja* Siebold & Zucc.) and *G. max* accessions actively produce BBI. However, BBI was absent in other *Glycine* species, including *Glycine curvata* Tindale, *Glycine cyrtoloba* Tindale, *Glycine latifolia* Benth., *Glycine microphylla* (Benth.) Tindale, *Glycine tabacina* (Labill.) Benth., and *Glycine tomentella* Hayata.

Soybean hull proteins Gly m 1.0101, Gly m 1.0102, and Gly m 2 are hydrophobic proteins that belong to the lipid transfer protein family and act as aeroallergens (Gonzalez et al., 1995). These proteins are unlikely to contribute to soybean‐based allergies, with KTI members being more associated with aeroallergen in soybean flour mills (P. Singh & Krishnaswamy, 2022).

Soybean profilin (Gly m 3) (Table S1) belongs to the profilin family, which is involved in the dynamic turnover and restructuring of the actin cytoskeleton. Gly m 3 was found to have some IgE‐binding activity in soybean‐sensitive people (Rihs et al., 1999). The binding activity appears to require the full length of the protein. This protein has high sequence homology with a birch (*Betula* species) pollen allergen, Bet v 2. The soybean protein starvation‐associated message 22 (SAM22)/(Gly m 4) (*Glyma.07g243651*) protein has high sequence homology with the birch pollen allergen Bet v 1 (Crowell et al., 1992). Most patients who reacted to Bet v 1 also have IgE binding in response to Gly m 4, with many patients who fall in this category also sensitive to other soy proteins (Mittag et al., 2004). Seed biotinylated proteins/(Gly m 7) (*Glyma.13g291800*, *Glyma.06g283900*, and *Glyma.06g283900*) were identified as having IgE binding capacity (Batista et al., 2007). They were later confirmed as an allergen that could induce a response in soybean allergic patients (Riascos et al., 2016). Gly m 7 may play a role in the plant as a source of biotin (Rajjou et al., 2012) and may also serve some purpose regarding desiccation (Battaglia et al., 2008). The allergenicity of the 2S albumin component (Gly m 8, previously Gly m 2S albumin) (*Glyma.12g213600* and *Glyma.13g288100*) was first described by Lin et al. (2006). After some conflicting studies, the 2S albumin component was later shown to possess strong antigens when presented to soybean‐allergic patients and was thus given the allergen designation of Gly m 8 (*Glyma.12g213600* and *Glyma.13g288100*) (Kattan & Sampson, 2015). 2S albumins have a conserved three‐dimensional structure, which imparts high stability to the gastrointestinal tract environment and high resistance to thermal processing (Ebisawa et al., 2013), hallmarks of a potential allergen.

#### 2.3.2 Engineering SSC for reduced allergenicity

Although P34 is not an abundant seed protein (<1%), it is present in most soybean genotypes (K. Bilyeu et al., 2009; C. Xu et al., 2007). A screening of 16,000 genotypes revealed only two genotypes, PI 567476 and PI 603570A, possessing a reduction in P34 (Joseph et al., 2006). These genotypes have a four‐bp insertion near the start codon, and molecular markers based on this mutation have been designed (K. Bilyeu et al., 2009). Alternatively, an RNAi‐based suppression of P34 can be employed as a means to reduce levels of the allergen in meals with minimal impact on other SSC characteristics (Herman et al., 2003; Schmidt et al., 2015).

With genome editing tools, it is now possible to remove or modify dominant allergens in soybean to create hypoallergenic genotypes. One example is the CRISPR/Cas9 creation of mutants for *lincCG1*, a long intergenic noncoding RNA (lincRNA) that controls the accumulation of β‐conglycinin (B. Song et al., 2024). The resulting *lincCG1* mutant lines lacked allergenic forms of β‐conglycinin (α′, α‐, and β), while also showing increased protein levels and higher amounts of free arginine and sulfur‐containing amino acids. Knocking out some gene family members of the β‐conglycinin and glycinin storage proteins/allergens (described as hypothetical mutant 1 in Figure 3d) will provide a new set of seed‐composition materials that can be analyzed for changes in allergenicity. It would be interesting to make mutations for multiple allergens (denoted as mutant 2 in Figure 3d) along with β‐conglycinin and glycinin by knocking out major KTI, BBI, P34, 2S albumins, and Gly m Bd 28K gene copies. For instance, CRISPR/Cas9 was used to generate BBI gene mutants, which resulted in significantly lower protease inhibitor content in soybean seeds (W. S. Kim et al., 2024). As most of these allergens also show protein rebalancing, it will be important to determine how the protein machinery in soybean seeds reacts to such concerted changes and the consequences of such changes in terms of allergenicity and other biological functions.

### 2.4 Carbohydrates

#### 2.4.1 Carbohydrates: Categories and distribution

Swine, poultry, and humans rely on the gut microbiome to properly digest feed and food, which can influence feed conversion and, thus, nutrition when consuming soybean‐based diet formulations (Parsons et al., 2000). Raffinose family oligosaccharides (RFOs), including both raffinose and stachyose, can reduce the amount of metabolizable energy in feed and cause flatulence and diarrhea, negatively impacting feed conversion (Coon et al., 1990; Suarez et al., 1999; Valentine et al., 2017). Thus, modifying CHO profiles has been an area of interest for soybean researchers as an avenue to develop superior meal composition.

CHOs constitute 30%–35% of seed weight and up to 40% of soybean meal after removing the oil (Chaudhary et al., 2015). Soybean CHOs are categorized into structural and non‐structural groups. The structural CHOs comprise dietary fiber components such as cell wall polysaccharides, non‐cellulose polysaccharides, and non‐CHO groups such as lignin and phenolic compounds (Middelbos & Fahey, 2008). In contrast, non‐structural CHOs are composed of low‐molecular‐weight (LMW) sugars, oligosaccharides, and storage polysaccharides (Karr‐Lilienthal et al., 2005; Middelbos & Fahey, 2008). Dietary fiber is highly variable and complex in structure. Primary nutritional fiber constituents in soybeans are cellulose, hemicellulose, pectins, and glycoproteins/proteoglycans, present primarily in cotyledons (Choct et al., 2010), while seed hulls also contain galactomannans, xylan hemicelluloses, uronic acids, and cellulose (Bittencourt et al., 2021).

**FIGURE 5:**
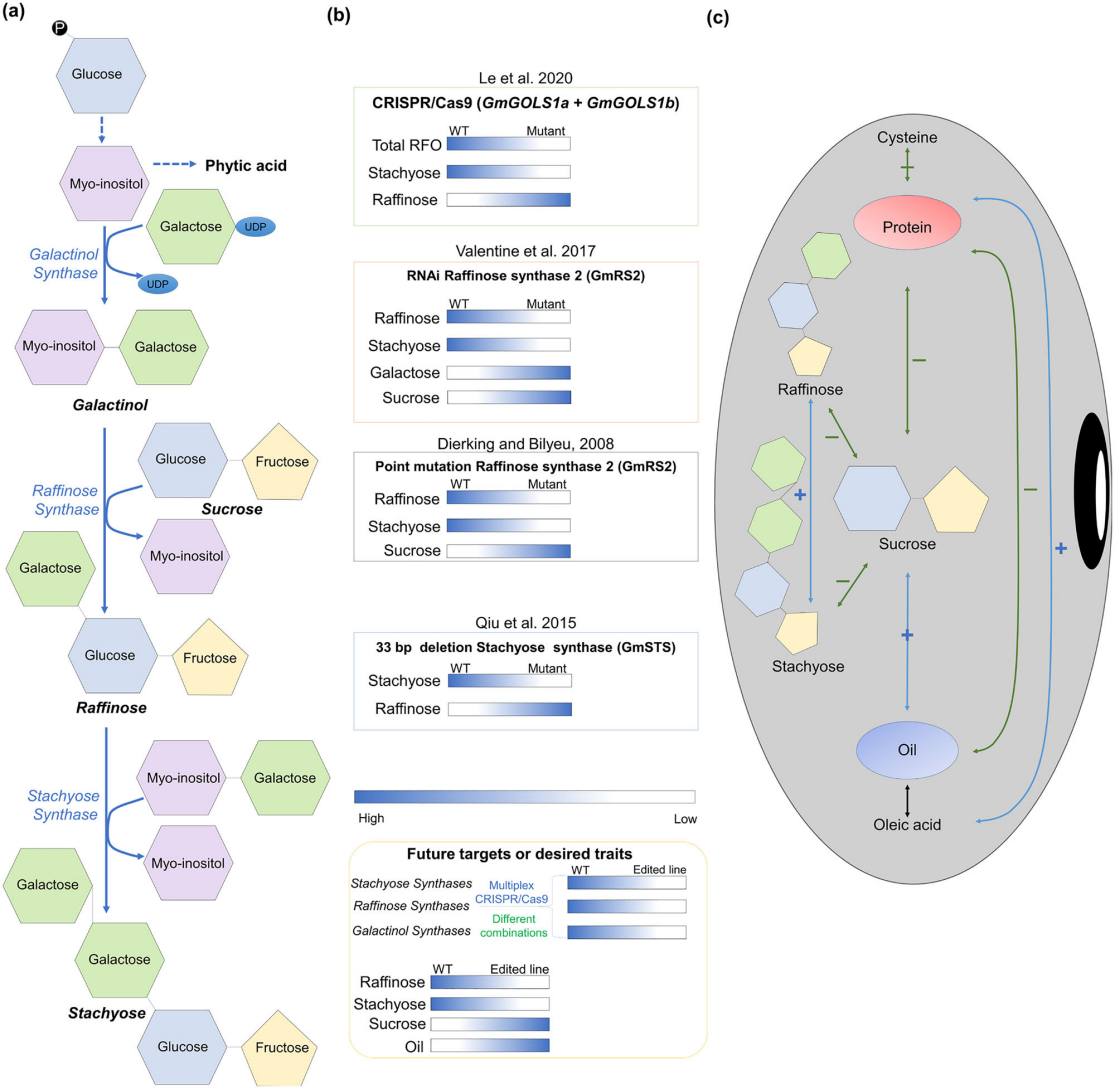
Raffinose family oligosaccharides (RFOs) biosynthesis and research updates in soybeans. (a) Biosynthetic pathway of RFOs in soybean. Sugars, predominantly sucrose, are imported to the developing cotyledons and converted to glucose‐6‐phosphate, which is used to make myo‐inositol, followed by galactinol, raffinose, and stachyose. Lines with dashes represent multiple‐step reactions, while bold lines represent single‐step reactions. Enzymes for the reactions are represented by blue text, while byproducts are indicated with black text. (b) Summary of some research done in the context of RFOs in soybean and future targets to achieve low raffinose, stachyose, and high sucrose (Dierking & Bilyeu, 2008; Hagely et al., 2020; Le et al., 2020; Qiu et al., 2015; Valentine et al., 2017). Proposed combinations of the above targets. (c) Correlation between oil, protein, and sugars. The blue line represents positive correlation, while the green lines represent negative correlation. For example, total oil and total sucrose are positively correlated with one another but negatively correlated with total protein. Correlations between cysteine and oleic acid are shown because they are important constituents of protein and oil, respectively.

Non‐structural CHOs represent half of the total CHOs in whole soybeans and meals (Karr‐Lilienthal et al., 2005). During processing, some sugars, including glucose, galactose, and fructose, are lost and not present in the final product. Important LMW sugars in soybean dry matter include sucrose, stachyose, raffinose, and verbascose, though their relative abundances can vary across genotypes and environments (Jo et al., 2019; Murai et al., 2024). The anti‐nutritional qualities of these oligosaccharides have motivated the development of low‐oligosaccharide genotypes, leading to the development of genotypes with 87% lower stachyose and raffinose concentrations (Dierking & Bilyeu, 2008; Parsons et al., 2000). The consequences of changes to other aspects of seed reserve production on oligosaccharides, including efforts to reduce lipid breakdown (Aznar‐Moreno et al., 2022; Kambhampati et al., 2021), have also been considered for their impact on oligosaccharide content. LMW sugars and storage polysaccharides (i.e., starches) are readily digestible by non‐ruminants (pigs, humans, and poultry) (Gdala et al., 1997; Marsman et al., 1997). Stachyose, raffinose, and verbascose are a type of oligosaccharide known as galacto‐oligosaccharides, containing terminal sucrose to which 1–3 galactose monomers are linked (Figure 5a). Together they make up ∼5% of whole soybean dry mass (DM) and 7%–8% of soybean meal (Grieshop et al., 2003; van Kempen et al., 2006). Current processing pipelines will not remove galacto‐oligosaccharides from meal. Using a genetic approach to reduce the level of galacto‐oligosaccharides may have pleiotropic effects on the plant, given these molecules play a role in both carbon transport and may function as protective agents during the maturation of drying seeds and cold stress challenge (Gilmour et al., 2000; Travert et al., 1997). However, there also exists evidence to the contrary to suggest plants will grow fine (Dierking & Bilyeu, 2009). Galacto‐oligosaccharides require α‐galactosidase for enzymatic digestion, which is absent in non‐ruminants.

#### 2.4.2 Carbohydrate rebalancing

A balance between each soluble sugar is maintained in the seed. For example, reducing stachyose may lead to an increase in other sugars, such as sucrose. Prior work indicates that late in development seeds receive exudates from the maternal plant; therefore, the production of oligosaccharides likely comes at the expense of existing reserves (Aznar‐Moreno et al., 2022; Kambhampati et al., 2021). The decreased stachyose, an anti‐nutritional factor, and increased sucrose may translate to a beneficial outcome for animal feed and human consumption, as higher sucrose levels impart more pleasant flavor characteristics to soy‐derived food products (Kumar et al., 2010; Saldivar et al., 2011) or could support biotechnological aims through a tradeoff that results in more oil (Aznar‐Moreno et al., 2022; Kambhampati et al., 2021). Key genes in soybean seed sugar production are listed in Table S2.

The composition of soybean seeds is determined by two key factors: the maternal supply of nutrients and their modification through various metabolic pathways during seed development (Allen & Young, 2013). In the later stages of seed development, both the quantity and concentration of exudates significantly decrease (Kambhampati et al., 2021). Concurrently, there is an increase in RFOs and insoluble components, which may contribute to a reduction in essential reserves such as oil and protein (Aznar‐Moreno et al., 2022; Kambhampati et al., 2021; Mukherjee et al., 2025).

Sebastian et al. (2000) analyzed bulk seed from 8000 M3 ethyl methane sulfonate (EMS) mutagenized soybean populations and the USDA germplasm collection for CHOs composition. From this screen, a single point mutation was observed in genotype PI 200508, leading to a 15% and 25% reduction in raffinose and stachyose concentrations, respectively, relative to other genotypes. PI 200508 also has a boost in sucrose content and a 50% reduction in phytic acid (Hitz et al., 2002). The genetic underpinnings of the observed seed traits are associated with a lesion in the *raffinose synthase* (*RS2*) (*Glyma.06G179200*) gene (Dierking & Bilyeu, 2008; Skoneczka et al., 2009). In another genotype survey, a 33 bp deletion in a putative *stachyose synthase* (*STS*) gene (*Glyma.19g217700*) was identified in PI 603176A that was associated with “ultra‐low” (0.5%) stachyose content and high sucrose content (Qiu et al., 2015). An identified EMS mutant of Williams 82 with lesions in *RS2* provides another strategy to achieve low raffinose levels (Dierking & Bilyeu, 2009). The biosynthesis pathway of RFOs in soybeans is shown in Figure 5a. As with other seed compositions, designing genetic strategies with the tools of biotechnology offers a route to create genotypes with favorable combinations of galactinol/raffinose/STSs to reduce the RFOs and increase the sucrose simultaneously (Figure 5b) (Dierking & Bilyeu, 2008; Hitz et al., 2002; Le et al., 2020; Qiu et al., 2015; Valentine et al., 2017). Furthermore, when strategies are taken to reduce potential supplies of carbon for oligosaccharides by limiting lipid degradation late in metabolism, the result is a positive influence on seed composition (Aznar‐Moreno et al., 2022).

Non‐structural polysaccharides like starch are primarily used for energy storage. Unlike other legumes such as beans (*Phaseolus vulgaris* L.), lentils (*Lens culinaris* Medik.), and peas (*Pisum sativum* L.), soybeans contain little starch, typically less than 3% of DM (Bednar et al., 2001). Starch varies among cultivars, ranging between 0.2% and 2.7% (Johnson et al., 2008), and typically contains more amylopectin than amylose (Stevenson et al., 2006), but as starch is transiently turned over when oligosaccharides are produced, it remains a target for engineering earlier in development (Kambhampati et al., 2021; Mukherjee et al., 2025).

### 2.5 Specialized metabolites: Basics and needs‐based engineering

#### 2.5.1 Saponins

Saponins are secondary metabolites in plants comprising a steroid or triterpenoid aglycone with varied CHO moieties connected by ether and ester linkages at different glycosylation sites (Berhow et al., 2006). The biting taste of soybeans is due to the presence of saponins. The complexity of saponin distribution across tissues is impacted by genotype and environment (Shimoyamada et al., 1990; Shiraiwa et al., 1991; Tsukamoto et al., 1995). Saponins are present in hypocotyls at higher levels relative to levels found in cotyledons and other plant parts. The variation in saponin composition in soybean seeds is partially explained by gene networks controlling the sugar chain length of soyasapogenol glycosides and soyasapogenol A (Sasama et al., 2010).

**FIGURE 6:**
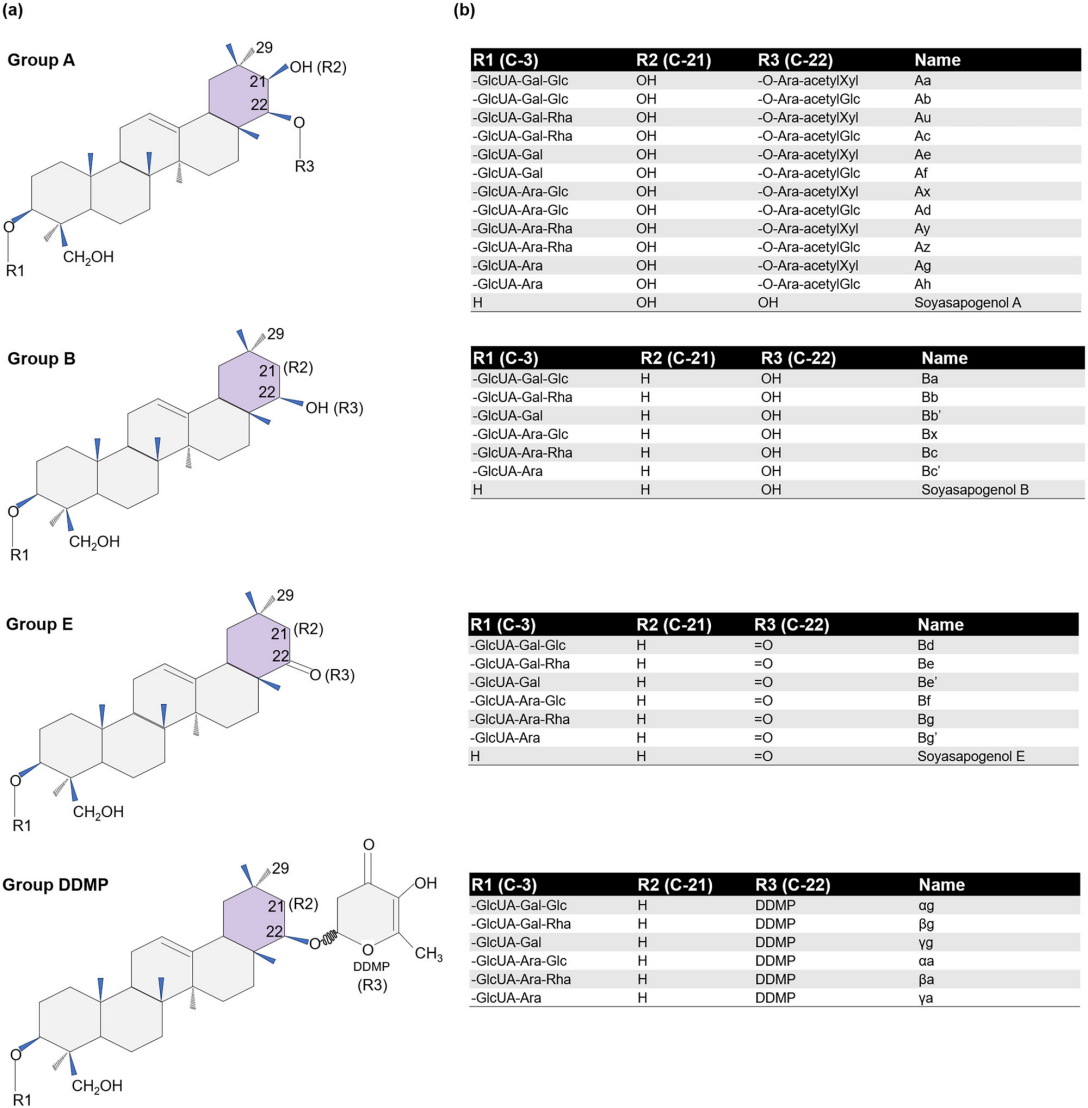
Different types of saponins in soybeans. (a) Saponins in soybean are categorized into groups A, B, E, and 2,3‐dihydro‐2,5‐dihydroxy‐6‐methyl‐4H‐pyran‐4‐one (DDMP). Their representative chemical structures are shown, and modifications are assigned as R1 at carbon 3 (C‐3), R2 at C‐21, and R3 at C‐22. The purple ring represents the ring with the most modifications on C‐21 and C‐22. (b) Adjacent tables for the respective saponin group show the modifications at different sites R1, R2, and R3 and subsequent names. Information was deduced from Tsuno et al. (2018).

The soybean saponins are mainly categorized into two groups on the basis of aglycone structures (Figure 6); group A includes bisdemoside saponins such as Soyasaponin aA_1_, Soyasaponin aA_2_, Soyasaponin aA_3_, Soyasaponin aA_4_, Soyasaponin aA_5_, Soyasaponin aA_6_, Soyasaponin aA_7_, and Soyasaponin aA_8_, and group B contains 2,3‐dihydro‐2,5‐dihydroxy‐6‐methyl‐4H‐pyran‐4‐one (DDMP) and monodesmoside saponins such as Soyasaponin I, Soyasaponin II, Soyasaponin III, Soyasaponin IV, Soyasaponin V, Soyasaponin βg, Soyasaponin βa, Soyasaponin γg, Soyasaponin γa, and Soyasaponin αg. (Berhow et al., 2006; Sundaramoorthy et al., 2018). Identification of 75 group A and 23 DDMP saponins was reported in nine soybean genotypes with diverse saponin patterns (Chitisankul et al., 2018). The structure of group A saponin is made up of two sugar side chains situated at the C‐3 and C‐22 hydroxyl groups on the triterpenoid aglycone (soyasapogenol A; 3β, 21β, 22β, 24‐tetrahydroxyolean‐12‐ene) (Sayama et al., 2012; Sundaramoorthy et al., 2018; Yano et al., 2017). This saponin group is the primary source of bitter flavor, resulting from the acetylation of the terminal sugar at the C‐22 position. Depending on the terminal sugar moieties, group A saponins are further categorized into four subgroups: Aa‐series, Ab‐series, A0‐series, and A‐series. The Aa‐ and Ab‐series appear to be controlled by a single gene designated *Sg‐1* (*Glyma.07g254600*) (Sayama et al., 2012; Takada et al., 2010) with separate co‐dominant *Sg‐1a* and *Sg‐1b* alleles controlling each series. A recessive allele designated as *sg‐1^0^* makes the terminal sugars glucose or xylose incapable of binding to the secondary sugars like arabinose at the C‐22 position, generating only A0 component, without group A acetyl saponin Aa‐ or Ab‐ series formed (Y. Takahashi et al., 2018).

The DDMP saponins contain one sugar moiety at the C‐3 hydroxyl position of aglycone (soyasapogenol B; 3β, 22β, 24‐trihydroxyolean‐12‐ene) and a DDMP moiety at the C‐22 position (Sayama et al., 2012; Sundaramoorthy et al., 2018; Yano et al., 2017). Extraction of group B and D saponins during processing results in the degradation of DDMP saponins (Kudou et al., 1992). The DDMP saponins have reduced bitterness and may possess health benefits, such as prevention of dietary hypercholesterolemia (Fenwick & Oakenfull, 1981), colon cancer cell proliferation repression (Ellington et al., 2005, 2006), and anti‐peroxidation of lipids and the liver‐protecting action of thyroid hormone secretion (Ishii & Tanizawa, 2006; Sasama et al., 2010). Additional saponin subcategories include groups B and E (Tsuno et al., 2018) that are derived from DDMP saponins during processing for food use and have similar health benefits as DDMP (Figure 6).

The Japanese genotype Tohoku No. 152 (T‐152) carries null mutations that prevent it from synthesizing both group A saponins and the three seed‐expressed lipoxygenase proteins. The mutation was traced to the *Sg‐5* locus (Sasama et al., 2010). In *G. soja*, group A saponin null mutants have been identified at the *Sg‐1* locus (Y. Takahashi et al., 2018). The genetic control of saponin biosynthesis is relatively complex. At least 11 alleles at five loci have been predicted as being involved in this process (Takagi et al., 2018). It appears that *Sg‐1* is the most important locus for knocking out just the bitter saponins. *Sg‐3* (possibly *Glyma.10g104700*) has a recessive allele that results in a knockout of “Ab” and “αg” saponins, which have a glucose moiety at the C‐3 position. Sg‐4 (*Glyma.01g046300*) was recently characterized (Takagi et al., 2018), in which a null allele (found in Williams 82, Enrei, Jack, and Suzuyutaka) failed to accumulate “Ad” and “βa” saponins. Recently, a hydroxylase gene, *Sg5* (*Glyma.15g243300*), was characterized, which also appears to reduce group A saponins (Sasama et al., 2010; Takada et al., 2013; Takagi et al., 2018). “B01082,” “ENT‐1376,” “R44,” “ENT‐1339,” and “S348P” all have mutations at the *Sg‐5* locus and exhibit little or no group A saponin accumulation (Yano et al., 2017). The loss‐of‐function mutants have no known adverse side effects (Sasama et al., 2010; Takada et al., 2013; Takagi et al., 2018; Yano et al., 2017). Thus, mutants lacking the group A saponins could have improved flavor profiles.

#### 2.5.2 Isoflavones

Isoflavones are a class of phytoestrogens, plant‐derived polyphenolic compounds with estrogen agonist or antagonist activity. Soybeans are a significant source of isoflavones consumed by humans, which have been linked with health benefits (Aldin et al., 2006; Artigot et al., 2013; Ma et al., 2015). However, isoflavones negatively impact the flavor and quality of soybean‐based food products (Aldin et al., 2006). The three primary isoflavones in soybean are genistein, daidzein, and glycitein (Table S3). Ma et al. (2015) showed glycitein is associated with negative quality characteristics in soymilk (bitter flavor, less sweetness, less smooth texture, poor color, and appearance). The soybean isoflavonoid biosynthesis pathway has not been entirely elucidated yet. Nonetheless, there is evidence that a *flavonoid 6‐hydroxylase* (*Glyma.08g326900*) activity is a key step in the synthesis of glycitein, which is supported by the observation that PI 567580A is a glycitein null genotype wherein the gene model for *Glyma.08326900* is not transcribed (Artigot et al., 2013).

#### 2.5.3 Tocopherols

Other minor compounds also contribute to the value of the seed composition, such as tocopherols, which play a role in oxidative stability of oils (Konda et al., 2019). Tocopherols are categorized as α, β, γ, and δ types (Sui et al., 2020). Several QTL controlling tocopherol level variations in soybean seeds have been identified (e.g., Knizia et al., 2022; Park et al., 2023; K. Yu et al., 2022; S. Zhang et al., 2023). The traditional refining, bleaching, and deodorization of soybean oil reduce the total tocopherol concentration in the refined oil to 800–1100 ppm. Tocopherols are naturally located in plastids and thylakoid membranes to protect against reactive oxygen species produced during photosynthesis. The key genes involved in the tocopherol biosynthesis pathway are listed in Table S4.

## 3 MODIFYING OIL COMPOSITION

### 3.1 The oil biosynthesis pathway

Soybean accounts for 59% of oilseed production worldwide (Soystats, 2024). Soybean oil is comprised of five different FAs, namely, linoleic acid (18:2), oleic acid (18:1), palmitic acid (16:0), linolenic acid (18:3), and stearic acid (18:0), with approximate shares of total FA content at 55%, 18%, 10%, 13%, and 4%, respectively (Figure 1a). The ratio of these FAs usually defines the oil quality for specific applications (Clemente & Cahoon, 2009). The abundance of unsaturated FAs (linoleic, oleic, linolenic) contributes to nutritional benefits. However, double bonds decrease oil stability, leading to oxidative breakdown and off‐flavors. Thus, high oleic (18:1) content is preferable to linolenic (18:3) for many applications (Clemente & Cahoon, 2009; Haun et al., 2014). Other FAs present at low levels (<1%) include arachidic (20:0) and behenic (22:0) (Allen et al., 2007).

**FIGURE 7:**
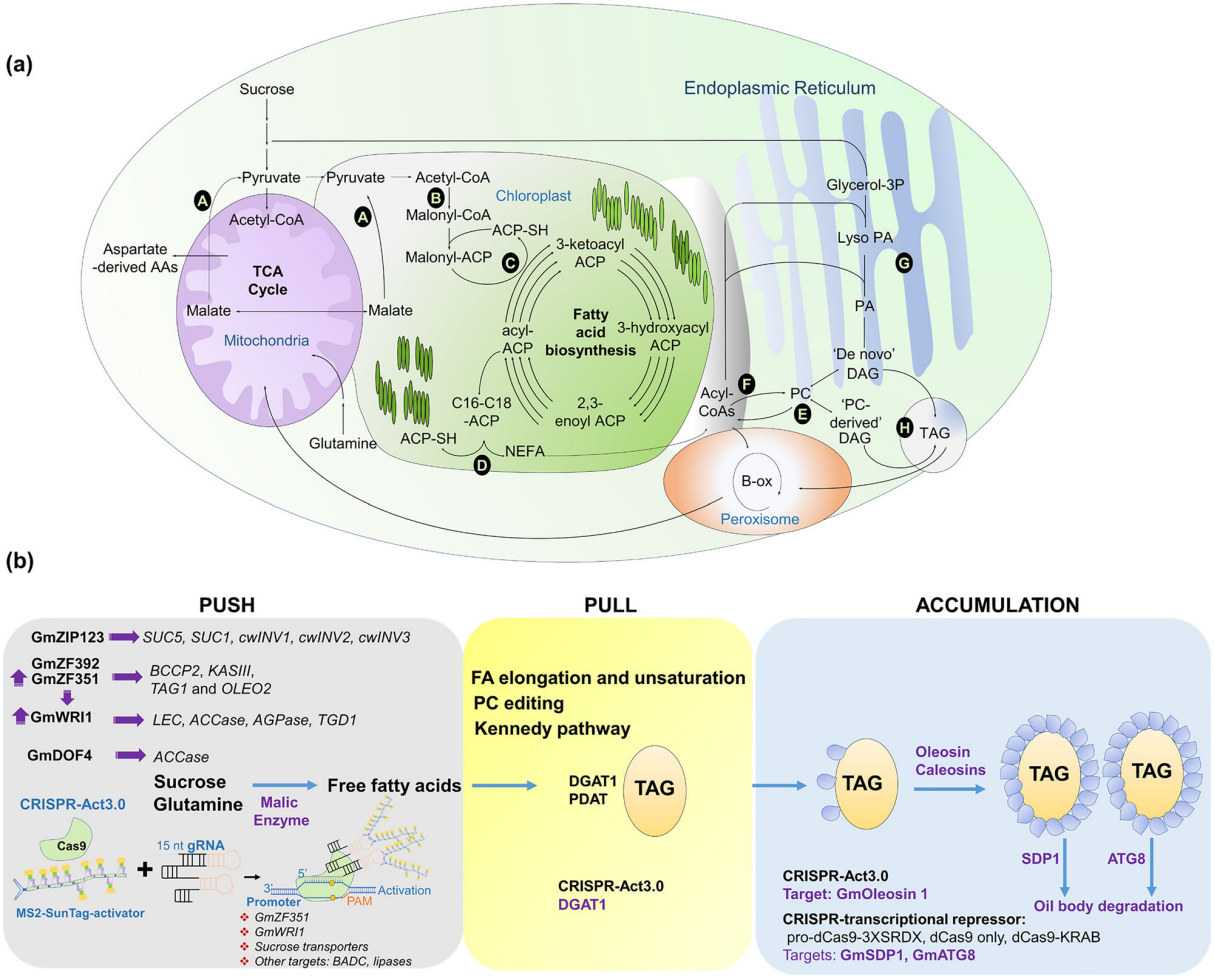
(a) The biosynthetic pathway of oil formation. A: Fatty acid de novo synthesis (FAS) begins in the chloroplast with pyruvate, which comes from sucrose and amino acid (AA) and organic acid metabolism, including malic enzyme. B: Acetyl‐CoA carboxylase converts acetyl‐CoA to malonyl‐CoA, followed by conversion to a malonyl‐acyl carrier protein (ACP). C: Malonyl‐ACP combines with an acyl‐ACP containing a sulfhydryl group (ACP‐SH) through ketoacyl synthase (KAS). D Fatty acid synthesis produces non‐esterified fatty acids (NEFA), which are exported from the chloroplast for incorporation into lipids using the eukaryotic pathway. E: In the endoplasmic reticulum, acyl editing results in the rapid exchange of acyl groups on and off phosphatidylcholine (PC) to increase the degree of unsaturation. F PC or acyl‐CoA binding proteins may be involved in the movement of acyl chains to the ER from the chloroplast. G: De novo glycerolipid assembly starts with the acylation of glycerol 3‐phosphate (G3P) to lysophosphatidic acid (Lyso PA) followed by a second acylation to phosphatidic acid (PA). H: Phosphatidic acid is hydrolyzed and a third acylation step results in triacylglycerol (TAG) formation, which takes place in the oleosome. (b) Transcriptional regulation of oil biosynthetic pathway genes. Increasing oil biosynthesis in soybean is possible by combining three main strategies: pushing fatty acid (FA) biosynthesis, pulling the free fatty acids (FFA) to TAG and finally, protecting the TAG from degradation (Allen, 2016a; Li‐Beisson et al., 2013; Sagun et al., 2023; Vanhercke et al., 2019; Z. Wang et al., 2022) and other references as indicated in the text.

In past decades, researchers have made systematic efforts to scrutinize the molecular mechanism of oil biosynthesis in soybeans. Oil biosynthesis in soybeans is categorized into four main stages: fatty acid de novo synthesis, acyl elongation and editing, triacylglycerol (TAG) assembly, and oil body formation (Bates et al., 2013). The FA synthesis is mainly confined to plastids, while TAG assembly and accumulation occur outside the plastid and are associated with the endoplasmic reticulum (ER) and the oil bodies (Figure 7). In soybean seeds, the primary carbon source, that is, pyruvate, initiates FA de novo synthesis. Pyruvate mainly comes from sucrose that is processed by glycolysis in soybeans (Mukherjee et al., 2025 and, to a more limited extent, from amino and organic acid metabolism using malic enzyme (Figure 7a‐A) (Allen & Young, 2013; Allen et al., 2009; Morley et al., 2023). Pyruvate derived from these sources is converted to acetyl‐CoA by pyruvate dehydrogenase.

The de novo fatty acid synthesis (FAS) pathway begins with acetyl‐CoA carboxylase (GmACCase), a heavily regulated step that serves as the entry point to FA synthesis and is often a rate‐limiting enzyme in the pathway (Figure 7a‐B). ACCase converts acetyl‐CoA to malonyl‐CoA, then further conversion to a malonyl‐acyl carrier protein (ACP) occurs by a transacylase. Malonyl‐ACP is combined with an existing acyl‐ACP through ketoacyl synthase (KAS) that elongates the acyl‐ACP followed by reduction, dehydration, and reduction steps that generate a series of acyl‐ACP intermediates as a part of FAS (Figure 7a‐C) (Jenkins et al., 2021; Li‐Beisson et al., 2017; Nam et al., 2020). The chain elongation cycle continues with KASI resulting in saturated 16‐carbon acyl‐ACP, which can either be hydrolyzed by GmFATB acyl‐ACP thioesterase or elongated by GmKASII with one more round of the FAS cycle to produce 18:0‐ACP (Shimakata & Stumpf, 1982). 18:0‐ACP can be desaturated to 18:1‐ACP by GmSACPD (Shanklin & Cahoon, 1998), which has been the subject of EMS mutagenesis for enhanced stearate content in soy (Jeong et al., 2018) or can be hydrolyzed by GmFATB generating 18:0 non‐esterified FAs. 18:1‐ACP is hydrolyzed by FATA thioesterase (Bates et al., 2013; Koo et al., 2004; Yang et al., 2019). During FA synthesis in the plastid, the 16:0 and 18:1 FAs are the main products. Their relative concentrations are affected by the enzymatic activities of FATA, FATB, 18:0‐ACP desaturase (SAD), and KASII (Figure 7a‐D) (Bates et al., 2013). The free FAs generated in the stroma of plastids are activated to acyl‐CoA that is used in lipid assembly. Importantly, although phosphatidylcholine is not inherently a component of the Kennedy pathway to produce TAG, it was observed by Bates and colleagues that phosphatidylcholine (PC) is the first labeled lipid from the supply of 14C‐acetate in soybeans (Bates et al., 2009). As PC is the substrate for desaturation of FAs in the (ER), the rapid exchange of acyl groups on and off PC can enhance the degree of unsaturation to levels that are present in TAG in soybeans, a phenomenon coined “acyl‐editing” (Figure 7a‐E) (Bates et al., 2009). Furthermore, it has been postulated that PC may provide a carrier molecule to move acyl changes through membrane contact to the endoplasmic reticulum possibly as an additional aspect of acyl editing prior to TAG production, which would further explain rapid PC labeling (Allen, 2016a; Tjellström et al., 2012). Mechanisms for acyl movement to the ER can also include acyl‐CoA binding proteins (Du et al., 2016) but remain an open question (Li‐Beisson et al., 2017; N. Li et al., 2016) (Figure 7a‐F).

The pathway for de novo glycerolipid assembly that is used for TAG biosynthesis from glycerol‐3‐phosphate and the acyl‐CoA pool is comprised of four enzymatic reactions minimally. The first enzymatic reaction involves the acylation of G3P by sn‐1 glycerol‐3‐phosphate acyltransferase followed by a second acylation with lysophosphatidic acid acyltransferase (Figure 7a‐G). Phosphatidic acid is hydrolyzed by phosphatidic acid phosphatase, and the third acylation is performed by diacylglycerol acyltransferase (DGAT) resulting in the TAG formation (Allen et al., 2015; Li‐Beisson et al., 2013). As this simplified description of TAG biosynthesis does not include desaturation steps, polyunsaturated fat synthesis requires desaturation of oleic acid (18:1) and linoleic (18:2) acyl chains while attached to phosphatidylcholine (PC) and involves conversions between diacylglycerol (DAG) and PC through multiple enzyme possibilities as summarized elsewhere (Allen, 2016a; Allen et al., 2015; Bates et al., 2013; Li‐Beisson et al., 2013). Linoleic acid is catalyzed by ω6‐desaturase (FAD2), of which two isoforms exist: one (FAD2‐1) found only in the seeds and the other (FAD2‐2) found in vegetative tissues and seeds. Linolenic acid is produced by the desaturation of 18:2 esterified to PC, which is catalyzed by FAD3A, FAD3B, and FAD3C. Reserve lipids (TAG) are stored in the cotyledon in oil bodies, also known as oleosomes or lipid bodies (Figure 7a‐H). A phospholipid monolayer covers their surface, embedded within proteins like oleosins, caleosins, and steroleosins (Frandsen et al., 2001).

### 3.2 Engineering seed oil quantity

Plant scientists have been working for decades to increase seed oil content. Final oil levels are a balance between the amount made and turned over during temporal development. Due to the availability of restricted maternal resources (Kambhampati et al., 2021; Pipolo et al., 2004), seed composition is dictated by the amount of resources it receives, along with the flux of these resources through biochemical pathways that leads to storage reserve production (Allen & Young, 2013). Temporal shifts in biomass composition during late seed maturation as observed in multiple oil crops, including arabidopsis and soybean, indicate partial turnover of lipids that result in less favorable reserve composition (Baud et al., 2008; Baud & Lepiniec, 2009; Chia et al., 2005; Kambhampati et al., 2021) and can include the oxidation of FAs (Koley et al., 2025) (Figure 7). Numerous efforts have been made to understand and explore the oil biosynthesis pathway in soybean and other oil crops to identify push, pull, package, and protect strategies that increase oil biosynthesis in seeds (Sagun et al., 2023; Vanhercke et al., 2019; Z. Wang et al., 2022) and leaves (K. L. Chu et al., 2022; Vanhercke et al., 2014). Briefly, this process involves pushing FA biosynthesis, pulling the free FA to TAG, and protecting the TAG from degradation (Figure 7). This strategy increased oil by up to 10% of total, with an increase in pyruvate‐derived amino acids and no negative impacts on total protein (Morley et al., 2023). This highlights how a single gene involved in central metabolism can be targeted to alter carbon partitioning for increased seed oil biosynthesis and, as described below, was the consequence of metabolic flux analyses. Pushing carbon toward acetyl‐CoA production for FAs in the plastids can be achieved by increasing the carbon source or increasing the activity of the enzymes involved in free FA biosynthesis.

#### 3.2.1 Increasing the carbon source

Sucrose availability is a limiting factor in FA biosynthesis. Until recently, few sugar transporters were known to affect SSC, particularly oil and protein contents and seed quality. Soybean contains 52 *GmSWEET* genes that encode sucrose transporters, categorized into distinct subfamilies (Patil et al., 2015). The *GmSWEET* gene family has shown diverse expression patterns, which suggest individualized roles in sugar transport during plant growth and in response to external stimuli.

*GmSWEET15* has been characterized as a sucrose transporter essential for embryo development by facilitating sucrose export from the endosperm to the embryo during early seed development (S. Wang et al., 2019). Further, the silencing of *GmSWEET15* showed a 60%–70% decrease in sucrose and caused seed abortion. Recently, GmSWEET39 was mapped as a key protein encoded by a QTL on chromosome 15 (H. Zhang et al., 2020). GmSWEET39 (soybean) and AtSWEET15 (arabidopsis) are orthologous pairs clustered with *GmSWEET15* and *AtSWEET11* and *12* in the same subfamily (Patil et al., 2015). A two‐nucleotide CC deletion that truncates the C‐terminus of *GmSWEET39* is associated with high‐seed oil and low‐seed protein (H. Zhang et al., 2020). Another set of sucrose transporters, *GmSWEET10a* and *GmSWEET10b*, also affect total seed oil/protein content and seed size by affecting sugar distribution from the seed coat to the embryo (S. Wang et al., 2020).

The combined modification of sucrose transporters or other enzymes could be used to alter their activity and pull more sucrose into the developing seeds, thereby increasing the carbon source for free FA biosynthesis. However, overexpressing such genes with strong constitutive promoters may result in nonspecific expression of the transgene and result in trait penalty (S. Wang et al., 2019). Fine‐tuning the expression behavior of these genes may be critical for enhancing their activity, resulting in efficient and enhanced carbon influx in the seed.

#### 3.2.2 Converting carbon from sugars to acyl chains

The next step is to convert the increased carbon to non‐esterified FAs. Many genes in this pathway are known, like *GmBCCP2*, *GmKASI*, *GmKASII*, *GmTAG*, *GmLEC*, *GmACCase*, *GmAGPase*, and *GmTGD* (Dobbels et al., 2017; Duan et al., 2023; H. Song et al., 2023; Virdi et al., 2020) (Table S5). Several TFs regulate the expression of multiple genes simultaneously. TFs reportedly involved in the regulation of lipid biosynthesis consist of *GmDof4*, *GmDof11* (Q. Sun et al., 2018; H. W. Wang et al., 2007), *GmbZIP123* (Q. X. Song, Li, et al., 2013), *GmLEC1a/GmLEC1b* (D. Zhang et al., 2017), *GmLEC2* (Manan et al., 2017; D. Zhang et al., 2017), *GmWRI1a* (L. Chen et al., 2018; Z. Wang et al., 2022), *GmMYB73* (Y. F. Liu et al., 2014), *GmZF351* (Q. T. Li et al., 2017; J. Y. Liu, Li, et al., 2020; Lu et al., 2021), and *GmDREBL* (Y. Q. Zhang et al., 2016). Overexpression of these TFs resulted in the positive regulation of enzymes involved in FA biosynthesis, elongation, and storage (Figure 7b). GmZF351 is an intriguing candidate to engineer the oil biosynthesis pathway, as it modulates the expression of *GmBCCP2*, *GmKASIII*, *GmTAG1*, *GmOLEO2*, and *GmWRI1* (Lu et al., 2021). GmWRI1 works upstream of *GmLEC*, *GmACCAse*, *GmAGPase*, and *GmTGD1* to regulate their expression (L. Chen et al., 2018); however, details of the cellular impact require further effort and could benefit from metabolic flux analyses.

#### 3.2.3 Pulling FAs to TAG

Once the acyl chains are synthesized, they are used in membrane lipids, and in seeds, a majority are used to make TAG. Acyl chains are desaturated by acyl editing on PC and assembled onto lipids by the Kennedy pathway. Two enzymes that have received considerable attention that enable the “pull” to TAG are GmDGAT1 (Flyckt et al., 2024; Jing et al., 2021; Lardizabal et al., 2008; Roesler et al., 2016; Torabi et al., 2021; Y. Xu et al., 2021), which attaches an acyl chain from an acyl‐CoA to DAG to make TAG and GmPDAT1 (J. Y. Liu, Li, et al., 2020; J. Y. Liu, Zhang, et al., 2020), which converts DAG to TAG by combining DAG with an acyl chain donated by PC. Other enzymatic steps, such as the release of the phosphate by phosphatidate phosphohydrolases (B. Chen et al., 2025), show promise for increased oil. As TAGs accumulate, they bud off from the endoplasmic reticulum, surrounded by oleosin proteins (D. Zhang et al., 2019) that package lipids into oil body storage structures. Ectopic expression of oleosins by the CRISPR‐Act3.0 system could be one possible strategy to enhance stability of oil droplets as increased oleosin production is part of the push, pull, package, protect strategy (Vanhercke et al., 2017). Packaging may limit TAG breakdown, which has been more directly considered through genetic engineering. Suppression of *SDP1* in soybean increases oil without negatively impacting protein content (Aznar‐Moreno et al., 2022; Kanai et al., 2019) and has also been demonstrated to increase oil content in other species, such as arabidopsis (van Erp et al., 2014), rapeseed (*Brassica napus* L.) (Kelly et al., 2013), jatropha (*Jatropha curcas* L.) (M. J. Kim, Yang, et al., 2014), and *Physaria fendleri* (A. Gray) (Azeez et al., 2022). Lipases, including FA reducer genes from the GDSL family, are drawing attention in soybean for similar reasons (Liao et al., 2025). Other oil‐body degrading enzymes like ATG8 could also be targeted to enhance the stability of TAG. Knocking out these genes could be a straightforward strategy using CRISPR/Cas9, but the function of these genes has not yet been studied extensively. Possibly complications can be avoided using dCas9‐3XSRDX, dCas9 alone, or dCas9‐KRAB systems (Karlson et al., 2021; Z. Li, Xiong, et al., 2019; Lowder et al., 2015).

### 3.3 Engineering quality of seed oil

Increasing oil biosynthesis and modifying oil quality are two related but distinct aspirations. As stated above, the proportion of the five FAs determines the quality of soybean oil. Altering the proportion of soybean FAs has the potential to tailor the oil for cooking, nutrition, health, industrial, and biofuel applications. Some applications may benefit from reducing the proportion of polyunsaturated FAs, primarily linolenic acid, which has lower oxidative stability and can lead to odd flavors and shorter shelf life (Hudson & Hudson, 2021). The chemical‐based hydrogenation of soybean oil leads to trans fats that are not favorable for some culinary uses as they produce trans FAs associated with heart disease (Hu et al., 1997). To make foods with solid fats, such as margarine, without hydrogenation, soybean oil must be high in saturated FAs. In soybeans, the primary saturated fat is palmitic acid (16:0), which is oxidatively stable and has a high melting point but is also associated with health concerns, including high cholesterol (Hu et al., 1997). The adverse health effects of palmitic acid and hydrogenation have pushed interest in increasing the oleic acid concentration in soybean oil (Fehr, 2007). Soybean accessions have varied oil compositions, and soybean accessions with standing mutations have allowed the production of some designer oil compositions through conventional breeding and genetic/genome engineering approaches (Demorest et al., 2016; Do et al., 2019; Hudson & Hudson, 2021).

Soybeans with low polyunsaturated FAs like linolenic acid are another desired trait. The FAD3 enzyme (omega‐3 FA desaturase) converts linoleic acid to linolenic acid, but oxidation of linolenic acid in soybean oil chemically results in non‐favorable traits, such as off‐flavors and reduced shelf life of soybean oil. Hence, reducing linolenic acid is a target of soybean breeders resulting in the development of specific genetic stocks. Soybean oil comprised of less than 3% linolenic acid is defined as “low linolenic,” while soybean oil comprised of less than 1% linolenic acid is defined as “ultra‐low‐linolenic” (Fehr, 2007). *FAD3A* (*Glyma.14g194300*) is highly expressed among the three homologous genes (*FAD3A*, *FAD3B*, and *FAD3C*) in developing soybean seeds (K. D. Bilyeu et al., 2003). Stacking mutations of *FAD3A* with *FAD3B* and *FAD3C* resulted in soybean oil with <3% linolenic acid. The decrease in linolenic acid percentage varied according to the number of mutant *FAD3* genes combined with *FAD2‐1* mutants, *FAD2‐1A* allele severity, and the environment (K. Bilyeu et al., 2005, 2006, 2011; Demorest et al., 2016; Pham et al., 2012; Reinprecht et al., 2009).

High oleic soybean lines have been commercialized. Hudson and Hudson (2021) are projected to cover 22% of soybean acreage in the US Delta‐12 FA desaturase 2 (Δ12 FAD2) is the critical enzyme that catalyzes the conversion of oleic acid to linoleic acid (Fehr, 2007; Haun et al., 2014; Lee et al., 2012; Schlueter et al., 2007; Thapa et al., 2016). The soybean genome contains two classes (FAD2‐1 and FAD2‐2) of microsomal‐6 desaturase. Two FAD2‐1 desaturases, *FAD2‐1A* (*Glyma.10g278000*) and *FAD2‐1B* (*Glyma.20g111000*), are highly expressed during peak seed oil biosynthesis. Loss‐of‐function mutations in both of these genes resulted in higher oleic acid (Buhr et al., 2002; Demorest et al., 2016; Do et al., 2019; Hudson & Hudson, 2021; Lee et al., 2012; Schlueter et al., 2007; Tang et al., 2005; Ulmasov et al., 2012).

Many stable soybean varieties with high oleic/low linolenic acid (HOLL) have been introduced through genetic engineering and traditional breeding. Two genetically modified soybean varieties with reduced polyunsaturated oil, Plenish by Pioneer‐Dupont in 2014 (https://www.healthyoils.corteva.com/about/plenish.html) and Vistive Gold by Monsanto‐Bayer in 2018 (Ulmasov et al., 2012), are available in the market. Similarly, HOLL from the genome‐edited variety generated through transcription activator‐like effector nucleases technology has been released by Calyxt (https://calyxt.com/) (Demorest et al., 2016; Haun et al., 2014). Public sector breeders have produced high oleic germplasm with reduced polyunsaturated FA (named Soyleic) through traditional plant breeding techniques (Pham et al., 2010, 2011, 2012). Other efforts include mutations in *FAD2‐1A* and *FAD2‐1B* introgressed into Heinong51 (Nan et al., 2020) and TruSoya that carries the *FAD2* mutations without the *FAD3* mutations, resulting in a high oleic profile and approximately 8% linolenic acid. The TruSoya profile thus exhibits an approximately 1:1 ratio of omega‐6:omega‐3 FAs, which may provide dietary health benefits (Kimball Nill, personal communication, 2022).

Although low linolenic acid (alpha‐linolenic acid [ALA]) is a desired trait for the stability of oil by oxidation, it is also important for the human diet. Soybeans provide ALA (Amjad Khan et al., 2017), and genetic loci responsible for higher linolenic acid content have been identified by QTL mapping in *G. soja* (Ha et al., 2014). Notably, expression of the *PfFAD3‐1* gene from *Physaria fendleri* (Yeom et al., 2020) or the *AtFAD3* (Eckert et al., 2006) under the control of a seed‐specific promoter resulted in significantly higher ALA (18:3) levels in soybean.

### 3.4 Multi‐omics approaches for modifying oil quantity and quality

Increasing the quantity and quality of oil in SSC for human consumption and industrial uses will require more in‐depth research using multi‐omics approaches, central dogma (CD) process‐based research, and seed development‐based analyses. Recently, researchers have focused on these aspects and introduced new technologies or methods to identify the key regulators of oil biosynthesis and FA composition in soybean. TILLING‐by‐Sequencing^+^ (TbyS^+^) and comprehensive bioinformatic tools were introduced to identify mutations on a population‐wide scale in soybean (Lakhssassi, Zhou, et al., 2021). The mutants obtained in this study could be utilized to improve oil composition traits by breeding programs or mutation stacking by genome engineering. Using TbyS^+^, Lakhssassi, Lopes‐Caitar, et al. (2021) characterized the five members of the *GmFAD2‐2* subfamily. The positive effect of increasing soybean seed oleic acid was noticed in the identified mutations (Lakhssassi, Lopes‐Caitar, et al., 2021).

Further, the soybean TILLING mutants could be implemented in breeding programs to improve seed FA composition traits (or other types of SSC traits as well). An EMS‐induced mutant population was recently generated, and whole‐genome sequencing of 1044 mutant lines was carried out to characterize induced mutations (M. Zhang et al., 2022). About 21.5% of plants showed visual phenotypic variation in the M2 population, including leaf morphology, plant architecture, and seed shape. Such a mutant population would be of great interest to studying oil biosynthesis pathway genes and other SSC traits in soybeans. However, seeds from this mutant population cannot be exported outside of China, limiting the utility of this resource for the broader research community.

Recent studies utilizing novel approaches have added new information about the biology of SSC. A recent meta‐analysis and transcriptome profiling study revealed hub genes involved in SSC traits during seed development (Qi et al., 2018), and other studies have started to link metabolites, transcripts, and flux (L. Li et al., 2015). Furthermore, three‐dimensional genetic networks were recently constructed using a new approach, with findings relevant to improving soybean seed oil and identifying gene functions (J. Y. Liu, Li, et al., 2020). Yang et al. (2019) found that oil accumulation rates and gene expression levels showed dynamic patterns during soybean seed development. The candidate genes identified by Yang et al. (2019) broaden our understanding of soybean seeds' molecular basis for oil accumulation. Researchers have recently integrated several publicly available datasets to select candidate genes likely to be involved in soybean seed oil biosynthesis and regulation, providing targets for breeding programs looking to increase oil content and quality (Turquetti‐Moraes et al., 2022). The candidate genes identified could be targeted using the above approaches or engineered tools.

### 3.5 Metabolic flux analysis in soybean seed oil biosynthesis

Metabolic flux analysis (MFA) has been instrumental in quantitatively describing the use of pathways for the push of carbon into FAS. Significant reductant required for FAS can be generated through glycolysis (Allen et al., 2009; Allen & Young, 2013), reducing the need for nicotinamide adenine dinucleotide phosphate (NADPH) from the oxidative pentose phosphate pathway. This is important, as this can conserve available carbon by minimizing oxidative steps that release carbon dioxide positively affecting the carbon conversion efficiency (reviewed in Sagun et al., 2023). Additionally, MFA using 13C‐labeled glutamine in soybeans revealed that malate is a byproduct of multiple glutamine deamidations and conversion from 2‐oxoglutarate. Soybeans require large amounts of nitrogen supplied by glutamine to generate amino acids for protein with transamidation reactions, thus generating significant amounts of organic acids as co‐products that must be processed for other use such as lipids (Allen, 2016a). The malate is subsequently converted by malic enzyme and pyruvate dehydrogenase for production of reductant and carbon for FAS (Allen et al., 2009; Allen & Young, 2013). These findings were recently validated genetically through engineering increased malic enzyme flux in soybeans and specifically indicated the importance of subcellular location for the activity (Morley et al., 2023; Schwender, 2023). The overexpression of malic enzyme genes increased oil by 0.5%–2% of mature seed biomass (i.e., up to 10% change in oil), with an increase in pyruvate‐derived amino acids and no negative impacts on total protein (Morley et al., 2023). The studies highlighted how a single gene involved in central metabolism can be targeted to alter carbon partitioning for increased seed oil biosynthesis when subcellular location is part of the consideration. The suite of malic enzymes with different subcellular locations have been annotated in oilseeds (Gerrard Wheeler et al., 2005), and recently it was shown that malic enzyme can be activated by glutamine as a part of kinetic characterization (Badia et al., 2025). This class of soybean enzymes have differing impact over development (Gerrard Wheeler et al., 2016) and are a prime target for further soybean seed compositional changes (Badia et al., 2025; Morley et al., 2023).

## 4 EXPLORING CROSS‐TALK BETWEEN PROTEIN, OIL, AND SUCROSE

Soybean seed protein and oil have a negative correlation (Figure 5c) (Hymowitz et al., 1972; Kambhampati et al., 2019; Wilcox & Shibles, 2001; Qi et al., 2024), and the molecular mechanisms underlying this relationship are not well understood. Past breeding efforts for increased protein levels have generally resulted in reduced oil levels and lower grain yields (Assefa et al., 2018; Bandillo et al., 2015; Chaudhary et al., 2015; Chung et al., 2003; de Mello Filho et al., 2004; Kambhampati et al., 2021; M. Kim et al., 2016; S. K. Singh et al., 2016). These reports indicate an inherent trade‐off between seed protein, oil, and yield in soybean. In Figure 5c, we show the generally positive and negative correlations for different seed components in soybean. A recent study demonstrated a novel approach to decouple the trade‐off between protein and oil or yield by optimizing nodulation number and improving nitrogen‐carbon assimilation (Zhong et al., 2024). Notably, two mutant lines of nodulation‐related *rhizobially induced cle1a/2a* (*ric1a/2a*) gene, generated using CRISPR/Cas technology, showed improved seed protein content, grain yield, and sustained oil content. Understanding and controlling the negative correlations may provide opportunities and strategies to develop designer soybeans for specific markets without compromising yields. There is a need for comprehensive studies of soybean seeds using mutants related to protein and oil composition, multi‐omics approaches/CD‐based research, and seed development‐based analysis to understand this trade‐off mechanism. Such studies would also benefit from isotope labeling and metabolic flux analyses (Allen, 2016b; Koley et al., 2024) that, as described above, continue to provide insights in soybeans (Allen et al., 2009; Allen & Young, 2013; Bates et al., 2009; Iyer et al., 2008; Mandy et al., 2014; Sriram et al., 2004) and provide cellular validation (Morley et al., 2023). Collaborative research efforts in these areas could reveal the mechanisms that govern the rebalancing and compensation of soybean seed constituents and alter the outcomes of these trade‐offs.

## 5 CHALLENGES AND FUTURE ASPECTS

Soybean is a highly valuable and widely grown crop due to its high yield and superior protein quality. Soybean seed, however, also contains many anti‐nutritional factors and allergens that reduce its overall value. Many important questions pertaining to the genetic control and biochemical mechanisms underlying the accumulation and re‐balancing of seed constituents remain. Seed protein content, oil content, and grain yield are critical traits in soybean, as are other parameters, such as allergens, saponins, sucrose, and raffinose. All of these traits require attention from researchers. Due to the complexity of the soybean genome, many important questions remain unanswered with respect to these traits.

There are ongoing efforts to address these challenges using both traditional breeding and genetic engineering strategies. Underlying this topic is the emergence of new engineering platforms with increased functional capacities. For example, a highly robust CRISPR‐Act3.0 system was recently developed that efficiently works in multiple crops like rice (*Oryza sativa* L.), arabidopsis, and tomato (*Solanum lycopersicum* L.) (Pan et al., 2022, 2021). CRISPR‐Act3.0 uses different strategies to recruit effectors and several transcription activators using a deactivated *Streptococcus pyogenes* Cas9 (dSpCas9). This system resulted in four‐fold to six‐fold higher transcriptional activation than the advanced CRISPR‐based activation systems. In addition, the tRNA‐gR2.0 (single guide RNA 2.0) expression system enabled CRISPR‐Act3.0‐based activation of up to seven genes for metabolic engineering in rice. Such combinatorial CRISPR‐based approaches could be tested in soybean to target the upstream region of transcription factors or directly modify the promoters of the genes of interest. This approach could be incorporated toward many of the SSC modification goals and targets discussed in this review.

Increasing soybean yields while maintaining the desired SSC traits is an important goal in improving soybean varieties. Many factors contribute to soybean yields, and one key factor is seed size, which can be influenced by cell size or number. Exploring the genetic factors that control seed size could be a fascinating research area, as it can potentially lead to the development of soybean varieties with larger seeds and higher yields. An example includes the DNA demethylase GmDMEa, which demethylates AT‐rich transposable elements involved in the activation of transcription factors and genes associated with the abscisic acid pathway, responsible for reduced seed size (W. Wang et al., 2024). CRISPR/Cas‐mediated knockout of *GmDMEa* resulted in larger seeds and increased yields without impacting the overall SSC content.

Combining traditional breeding with new and efficient genetic engineering tools, multi‐omics approaches, and emerging technologies (e.g., machine learning, single‐cell transcriptomics, single‐cell metabolomics, metabolic flux analyses, and single‐cell proteomics) will be critical for the soybean community to meet future demands in terms of product quantity and quality. Recent and emerging developments promise to accelerate the pace of engineered/edited trait introgression into breeding programs and will be significant assets to characterize the biological mechanisms related to SSC.

## AUTHOR CONTRIBUTIONS

**Ritesh Kumar**: Conceptualization; visualization; writing—original draft; writing—review and editing. **Steven Mulkey**: Conceptualization; writing—review and editing. **Rahul Mahadev Shelake**: Conceptualization; writing—review and editing. **Rachel Combs‐Giroir**: Writing—review and editing. **Thiya Mukherjee**: Writing—review and editing. **Doug K. Allen**: Writing—review and editing. **Tom Elmo Clemente**: Writing—review and editing. **Minviluz G. Stacey**: Writing—review and editing. **Aaron J. Lorenz**: Writing—review and editing. **Robert M. Stupar**: Conceptualization; supervision; writing—review and editing.

## CONFLICT OF INTEREST STATEMENT

Rahul Mahadev Shelake, Doug K. Allen, Tom Elmo Clemente, Minviluz G. Stacey, and Robert M. Stupar are co‐inventors on various respective patents concerning plant biotechnology methods and traits. Ritesh Kumar is currently employed by a company with research and development efforts in the plant biotechnology space. The remaining authors declare no conflicts of interest.

## Supporting information




**Table S1**. Soybean gene models encoding seed protein storage / allergens / anti‐nutritional factors.
**Table S2**. Soybean gene models putatively involved in seed sugar production.
**Table S3**. Soybean gene models putatively involved in flavor characteristics.
**Table S4**. Soybean gene models putatively involved in the tocopherol biosynthesis pathway.
**Table S5**. Soybean gene models putatively involved in seed oil composition.

## Data Availability

There are no original data associated with this article. Data reviewed in this article are summarized in the figures and supplemental tables. Referenced data are available in the cited literature.
