# Supplementary material for: Targets and strategies to design soybean seed composition traits
Source: Plant Genome. 2025 Sep 26;18(4):e70115. doi: 10.1002/tpg2.70115 (PMC12475100; doi:10.1002/tpg2.70115)
Supplement: Supplementary file 1 — Table S1. Soybean gene models encoding seed protein storage / allergens / anti‐nutritional factors. Table S2. Soybean gene models putatively involved in seed sugar production. Table S3. Soybean gene models putatively involved in flavor characteristics. Table S4. Soybean gene models putatively involved in the tocopherol biosynthesis pathway. Table S5. Soybean gene models putatively involved in seed oil composition. [file TPG2-18-e70115-s001.pdf]

Table S1. Soybean gene models encoding seed protein storage / allergens / anti-nutritional factors

Molecular	Gene symbol	Chromosome	Wm82.a1.v1	Wm82.a1.v1.1	Wm82.a2.v1	Wm82.a4.v1	Start Position (Wm82.a2.v1)	Stop Position (Wm82.a2.v1)	Comments
Kunitz Trypsin Inhibitor	*KTI1*	Gm01	Glyma01g10900	Glyma01g10900	Glyma.01g095000	Glyma.01g095000	29302459	29303779	one of three primary KTI loci based on literature
Kunitz Trypsin Inhibitor	.	Gm01	Glyma01g11640	Glyma01g11640	Glyma.01g096200	Glyma.01g096200	30592394	30593278	KTI locus based on gene homology
Kunitz Trypsin Inhibitor	.	Gm06	Glyma01g11870	Glyma01g11870	Glyma.06g219900	Glyma.01g095232	25165353	25166156	KTI locus based on gene homology
Kunitz Trypsin Inhibitor	.	Gm08	Glyma08g25520	Glyma08g25520	Glyma.08g235400	Glyma.08g235400	19781264	19782288	KTI locus based on gene homology
Kunitz Trypsin Inhibitor	.	Gm08	Glyma08g45490	Glyma08g45490	Glyma.08g341100	Glyma.08g341100	45715456	45716332	KTI locus based on gene homology
Kunitz Trypsin Inhibitor	*KTI2/KTIS*	Gm08	Glyma08g45520	Glyma08g45520	Glyma.08g341400	Glyma.08g341400	45728546	45729738	one of three primary KTI loci based on literature
Kunitz Trypsin Inhibitor	*KTI3*	Gm08	Glyma08g45530	Glyma08g45531	Glyma.08g341500	Glyma.08g341500	45733267	45736194	one of three primary KTI loci based on literature
Kunitz Trypsin Inhibitor	.	Gm08	Glyma08g45540	Glyma08g45540	Glyma.08g341600	Glyma.08g341600	45739567	45740774	KTI locus based on gene homology
Kunitz Trypsin Inhibitor	.	Gm08	Glyma08g45550	Glyma08g45550	Glyma.08g341700	Glyma.08g341700	45742856	45743690	KTI locus based on gene homology
Kunitz Trypsin Inhibitor	.	Gm08	Glyma08g45560	Glyma08g45560	Glyma.08g341800	Glyma.08g341800	45745300	45746386	KTI locus based on gene homology
Kunitz Trypsin Inhibitor	.	Gm08	Glyma08g45580	Glyma08g45580	Glyma.08g342000	Glyma.08g342000	45759318	45762185	KTI locus based on gene homology
Kunitz Trypsin Inhibitor	.	Gm08	Glyma08g45590	Glyma08g45590	Glyma.08g342100	Glyma.08g342100	45767848	45768822	KTI locus based on gene homology
Kunitz Trypsin Inhibitor	.	Gm08	Glyma08g45610	Glyma08g45610	Glyma.08g342300	Glyma.08g342300	45784666	45785600	KTI locus based on gene homology
Kunitz Trypsin Inhibitor	.	Gm18	Glyma18g41540	Glyma18g41540	Glyma.18g191400	Glyma.18g191400	46197776	46198579	KTI locus based on gene homology
Bowman-Birk Proteinase Inhibitor	.	Gm09	Glyma09g28700	Glyma09g28700	Glyma.09g158500	Glyma.09g158500	38186782	38187428	Bowman-Birk serine protease inhibitor family
Bowman-Birk Proteinase Inhibitor	.	Gm09	no correspondence	Glyma09g28706	Glyma.09g158600	Glyma.09g158600	38193356	38193892	Paralog of Glyma.16g108900. "Bowman-Birk serine protease inhibitor family"
Bowman-Birk Proteinase Inhibitor	.	Gm09	no correspondence	Glyma09g28713	Glyma.09g158700	Glyma.09g158700	38198232	38198735	Bowman-Birk serine protease inhibitor family
Bowman-Birk Proteinase Inhibitor	.	Gm09	Glyma09g28720	Glyma09g28720	Glyma.09g158800	Glyma.09g158800	38203636	38203887	Bowman-Birk serine protease inhibitor family
Bowman-Birk Proteinase	*BBI*	Gm09	Glyma09g28730	Glyma09g28730	Glyma.09g158900	Glyma.09g158900	38210078	38210416	THE primary BBI

Molecular	Gene symbol	Chromosome	Wm82.a1.v1	Wm82.a1.v1.1	Wm82.a2.v1	Wm82.a4.v1	Start Position (Wm82.a2.v1)	Stop Position (Wm82.a2.v1)	Comments
Inhibitor									
Bowman-Birk Proteinase Inhibitor	.	Gm09	Glyma09g39630	Glyma09g39630	Glyma.09g260400	Glyma.09g260400	47869785	47870229	Paralog of Glyma.18g231500. "Bowman-Birk serine protease inhibitor family"
Bowman-Birk Proteinase Inhibitor	.	Gm09	Glyma09g39640	Glyma09g39640	Glyma.09g260500	Glyma.09g260500	47871771	47872421	Bowman-Birk serine protease inhibitor family
Bowman-Birk Proteinase Inhibitor	.	Gm14	Glyma14g26400	Glyma14g26400	Glyma.14g117600	Glyma.14g117600	15524438	15524689	"Bowman-Birk serine protease inhibitor family"
Bowman-Birk Proteinase Inhibitor	.	Gm14	Glyma14g26410	Glyma14g26410	Glyma.14g117700	Glyma.14g117700	15530056	15530697	"Bowman-Birk serine protease inhibitor family"
Bowman-Birk Proteinase Inhibitor	.	Gm16	Glyma16g33400	Glyma16g33400	Glyma.16g208900	Glyma.16g208900	36838653	36839376	Paralog of Glyma.09g158600 . "Bowman-Birk serine protease inhibitor family"
Bowman-Birk Proteinase Inhibitor	.	Gm18	Glyma18g46550	Glyma18g46550	Glyma.18g231400	Glyma.18g231400	52007700	52008228	Bowman-Birk serine protease inhibitor family
Bowman-Birk Proteinase Inhibitor	.	Gm18	Glyma18g46560	Glyma18g46560	Glyma.18g231500	Glyma.18g231500	52019138	52019828	Paralog of Glyma.09g260400. "Bowman-Birk serine protease inhibitor family"
Bowman-Birk Proteinase Inhibitor	.	Gm18	Glyma18g46580	Glyma18g46580	Glyma.18g231700	Glyma.18g231700	52031400	52031909	
P34 Allergen (paralog)	.	Gm05	Glyma05g29130	Glyma05g29131	Glyma.05g158600	no correspondence	35068575	35070218	Paralog to the P34 allergen gene
β-Congylcinin α-subunit (2)	*CG-3*	Gm20	Glyma20g28660	Glyma20g28660	Glyma.20g148300	Glyma.20g148300	38678459	38680997	
β-Congylcinin α-subunit (1)	*CG-2 / Cgy2 / CG-ALPHA-2*	Gm20	Glyma20g28650	Glyma20g28650	Glyma.20g148400	Glyma.20g148400	38684625	38687183	Associated protein described on soybase as an 'alpha prime subunit 2…"
β-Congylcinin α'-subunit (1)	*CG-1 / Cgy1 / GM7S*	Gm10	Glyma10g39150	Glyma10g39150	Glyma.10g246300	Glyma.10g246300	47483367	47486096	alpha prime subunit 1 (listed as a paralog to Glyma20g28650 (alpha subunit) on v1 of soybase, and Glyma.20g148300 (the other alpha subunit) on v2)
β-Congylcinin α'-subunit (2)	*Cgy1 / GM7S*	Gm10	Glyma10g39170	Glyma10g39170	Glyma.10g246500	Glyma.10g246500	47495824	47498147	beta chain-like
β-Congylcinin α'-subunit (3)	.	Gm20	no correspondence	Glyma20g28466	Glyma.20g146300	no correspondence	38475899	38478861	Paralog of (Glyma.10g246500). Possibly the alpha' subunit psuedo-gene on GM20
β-Congylcinin β-	*Cgy4*	Gm20	Glyma20g28460	Glyma20g28460	Glyma.20g146200	Glyma.20g146200	38469427	38471651	Associated protein described

Molecular	Gene symbol	Chromosome	Wm82.a1.v1	Wm82.a1.v1.1	Wm82.a2.v1	Wm82.a4.v1	Start Position (Wm82.a2.v1)	Stop Position (Wm82.a2.v1)	Comments
subunit (1)									on soybase as the 'beta-subunit 4'
β-Congylcinin β-subunit (2)	.	Gm20	Glyma20g28640	Glyma20g28640	Glyma.20g148200	Glyma.20g148200	38662515	38664740	
β-Congylcinin β-subunit (3)	.	Gm10	no correspondence	no correspondence	no correspondence	no correspondence	47487929	47495178	Paralog to (Glyma.20g148200) in v2 only
β-Congylcinin null allele	*Sgc-1*	Gm20					.	.	Deletion between Glyma.20g148300 and Glyma.148400, leaving just 197 bp between the two
Glycinin subunit G1	*Gy1*	Gm03	Glyma03g32030	Glyma03g32030	Glyma.03g163500	Glyma.03g163533	37817334	37825976	
Glycinin subunit G2	*Gy2*	Gm03				Glyma.03g163467	39830110	39837209	
Glycinin subunit G3	*Gy3*	Gm19	Glyma19g34780	Glyma19g34780	Glyma.19g164900	Glyma.19g164900	42567390	42570310	
Glycinin subunit G4	*Gy4*	Gm10	Glyma10g04280	Glyma10g04280	Glyma.10g037100	Glyma.10g037100	3256351	3259176	
Glycinin subunit G5	*Gy5*	Gm13	Glyma13g18450	Glyma13g18450	Glyma.13g123500	Glyma.13g123500	23665940	23668679	
Glycinin subunit	*Gy6*	Gm03					39830367	39833159	DOESN'T EXIST IN VERSION 2 ASSEMBLY… (Distance is based on v1 assembly…)
Glycinin subunit	*Gy7*	Gm19	Glyma19g34770	Glyma19g34770	Glyma.19g164800	Glyma.19g164800	42559312	42561641	Referred to as a paralog of Glyma03g32020 in the v1 assembly
SAM22 (aka *Gly m 4*)	.	Gm07	Glyma07g37240	Glyma07g37240	Glyma.07g243500	Glyma.07g243500	42331425	42332476	Also known as PR-10 (pathogenesis related, in the Bet v I family from Birch pollen)
SAM22 (aka *Gly m 4*) (homology)	.	Gm17	no correspondence	Glyma17g03365	Glyma.17g030400	Glyma.17g030400	2222144	2223193	Paralog of (Glyma.07g243500)
Profilin (aka *Gly m 3*)	.	Gm01	Glyma01g00920	Glyma01g00920	Glyma.01g005900	Glyma.01g005900	576688	580396	Annotated as 'Profilin 4'
Profilin (aka *Gly m 3*)	.	Gm03	Glyma03g40990	Glyma03g40990	Glyma.03g249600	Glyma.03g249600	44569709	44572013	Annotated as "Profilin", or "Profiin family protein"
Profilin (aka *Gly m 3*)	.	Gm05	Glyma05g35980	Glyma05g35980	Glyma.05g225600	Glyma.05g225600	40427955	40429788	Annotated as "Profilin 5"
Profilin (aka *Gly m 3*)	.	Gm05	Glyma05g35970	Glyma05g35970	Glyma.05g225700	Glyma.05g225700	40432149	40439631	Annotated as "Profilin 2"
Profilin (aka *Gly m 3*)	.	Gm07	Glyma07g15090	Glyma07g15090	Glyma.07g125700	Glyma.07g125700	15014654	15017077	Paralog of (Glyma.01g005900), annotated as "Profilin 4"
Profilin (aka *Gly m 3*)	.	Gm08	Glyma08g03640	Glyma08g03640	Glyma.08g032400	Glyma.08g032400	2591815	2593508	Paralog of (Glyma.05g225600), annotated as "profilin 5"

Molecular	Gene symbol	Chromosome	Wm82.a1.v1	Wm82.a1.v1.1	Wm82.a2.v1	Wm82.a4.v1	Start Position (Wm82.a2.v1)	Stop Position (Wm82.a2.v1)	Comments
Profilin (aka *Gly m 3*)	.	Gm08	Glyma08g03650	Glyma08g03650	Glyma.08g032500	Glyma.08g032500	2596659	2598736	Paralog of (Glyma.05g225700), annotated as "profilin 2"
Profilin (aka *Gly m 3*)	.	Gm08	Glyma08g03660	Glyma08g03660	Glyma.08g032600	Glyma.08g032600	2602434	2603938	Annotated as "profilin 4" and "profilin 5". No paralogs.
Profilin (aka *Gly m 3*)	.	Gm15	Glyma15g02380	Glyma15g02380	Glyma.15g020700	Glyma.15g020700	1603915	1605757	Annotated as "profilin 4". No paralogs.
Profilin (aka *Gly m 3*)	.	Gm19	Glyma19g43640	Glyma19g43640	Glyma.19g247100	Glyma.19g247100	49354909	49361144	Paralog of (Glyma.19g247100), annotated as "Profilin", or "Profiin family protein"
P22-25	.	.					.	.	.
Lectin (agglutinin)	*Le1-1*	Gm02	Glyma02g01590	Glyma02g01590	Glyma.02g012600	Glyma.02g012600	1123507	1125658	Annotated as "Lectin 1 gene 1".
Lectin (agglutinin)	*Le2-1*	Gm10	Glyma10g01620	Glyma10g01620	Glyma.10g013100	no correspondence	1176602	1177408	Paralog of (Le1-1). Annotated as "Lectin 2 gene 1".
Lipoxygenase	*Lx2* or *LOX2* or *LOX1.2*	Gm13	Glyma13g42310	Glyma13g42310	Glyma.13g347500	Glyma.13g347500	43761727	43766023	No paralog.
Lipoxygenase	*Lx1* or *LOX1.1*	Gm13	Glyma13g42320	Glyma13g42320	Glyma.13g347600	Glyma.13g347600	43769021	43773290	No paralog.
Lipoxygenase	*Lx3* or *LOX3* or *LOX1.3*	Gm15	Glyma15g03030	Glyma15g03030	Glyma.15g026300	Glyma.15g026300	2123754	2128104	No paralog.
Seed Biotinylated Protein (*Gly m 7*)	.	Gm13	Glyma13g36780	Glyma13g36780	Glyma.13g291800	Glyma.13g291800	39190331	39193461	Based on the cDNA sequence, the gene maps (with fidelity) to pos: 39191097 to 39191794 on chromosome 13. Gene spanning where the sequence maps to is annotated as a 'late embryogenesis abundant (LEA) protein'. Jackpot.
Seed Biotinylated Protein (*Gly m 7*)	.	Gm12	Glyma12g33710	Glyma12g33711	Glyma.12g209500	Glyma.12g209500	36869301	36872356	Possible paralog based on sequence homology (second highest sequence fidelity). Maps to pos: 36871123 to 36871814. Gene spanning where this sequence maps to is annotated as 'late embryogenesis abundant (LEA) protein" based on arabidopsis. Soybase lists this gene as a paralog of (Glyma.13g291800). Jackpot.
Seed Biotinylated Protein (*Gly m 7*)		Gm06	Glyma06g43640	Glyma06g43641	Glyma.06g283900	Glyma.06g283900	47201931	47206514	A small portion of the cloned gene mapped to pos. 47205906 to 47205946 on

Molecular	Gene symbol	Chromosome	Wm82.a1.v1	Wm82.a1.v1.1	Wm82.a2.v1	Wm82.a4.v1	Start Position (Wm82.a2.v1)	Stop Position (Wm82.a2.v1)	Comments
									chromosome 6. This region is spanned by a gene which has been annotated as a "late embryogenesis abundant (LEA) domain containing protein" based on arabidopsis. Interesting. No paralogs.
2S Albumin (*Gly m 8*)	.	Gm13	Glyma13g36400	Glyma13g36400	Glyma.13g288100	Glyma.13g288100	38876942	38877822	The cDNA for this gene mapped to a region spanned by this glyma gene model, which has been annotated as having "nutrient reservoir activity, protease inhibitor", and overlapped with 'published genes' for albumin
2S Albumin (*Gly m 8*)		Gm12	Glyma12g34160	Glyma12g34160	Glyma.12g213600	Glyma.12g213600	37245953	37246701	Paralog of (Glyma.13g288100), annotated as having "protease inhibitor, seed storage" activity. Also covers the other region to which the cDNA for the allergen gene mapped to.
Phytic Acid	*LPA1*	Gm03	Glyma03g32500	Glyma03g32500	Glyma.03g167800	Glyma.03g167800	38202353	38210883	This gene is a homolog of the maize lpa1 gene, encoding an ABC transporter that may be involved in phytate partitioning. A recessive deletion at this locus led to a significant decrease in phytic acid in a mutant line derived from CX1834 (Wilcox et al., 2000; Gillman et al., 2009)
Phytic Acid	*LPA2*	Gm19	Glyma19g35230	Glyma19g35230	Glyma.19g169000	Glyma.19g169000	42961519	42970330	This gene is a homolog of the maize lpa1 gene, encoding an ABC transporter that may be involved in phytate partitioning. A recessive deletion at this locus led to a significant decrease in phytic acid in a mutant line derived from CX1834 (Wilcox et al., 2000; Gillman et al., 2009). The deletion at THIS locus is not as effective as the one on Gm3, but they do stack additively.

Molecular	Gene symbol	Chromosome	Wm82.a1.v1	Wm82.a1.v1.1	Wm82.a2.v1	Wm82.a4.v1	Start Position (Wm82.a2.v1)	Stop Position (Wm82.a2.v1)	Comments
Phytic Acid	*MIPS1*	Gm11	no correspondence	no correspondence	Glyma.11g238800	Glyma.11g238800	33319226	33322244	Myo-inositol phosphate synthase. Catalyzes the first step in the synthesis of phytic acid. A mutation at this gene was found to reduce the proportion of phytic acid relative to total P down to 50% (Hitz et al. 2002; Saghai-Maroof et al., 2009)
Phytic Acid	*MIPS2*	Gm18	Glyma18g02210	Glyma18g02210	Glyma.18g018600	Glyma.18g018600	1365145	1368075	Paralog of MIPS1 (Glyma.11g238800)
Phytic Acid	*MIPS3*	Gm05	Glyma05g31450	Glyma05g31450	Glyma.05g180600	Glyma.05g180600	36870768	36875196	The Genebank entry described as MIPS3 (DQ323907) BLASTED to several linkage groups, including the MIPS1 and MIPS2, and MIPS4 loci.
Phytic Acid	*MIPS4*	Gm08	Glyma08g14670	Glyma08g14670	Glyma.08g138200	Glyma.08g138200	10592507	10597043	Paralog of MIPS3 (Glyma.05g180600).

Table S2. Soybean gene models putatively involved in seed sugar production

Raffinose-Stachyose Synthesis Pathway

Molecular	Gene symbol	Chromosome	Wm82.a1.v1	Wm82.a1.v1.1	Wm82.a2.v1	Wm82.a4.v1	Start Position (Wm82.a2.v1)	Stop Position (Wm82.a2.v1)	Comments
Stachyose Synthase	.	Gm19	Glyma19g40550	Glyma19g40550	Glyma.19g217700	Glyma.19g217700	47033812	47037286	aka galactinol:raffinose 6-galactosyltransferase. Catalyzes synthesis of stachyose from raffinose and galactinol.
Raffinose Synthase	.	Gm02	Glyma02g47330	Glyma02g47330	Glyma.02g303300	Glyma.02g303300	47864899	47869138	aka galactinol:sucrose 6-galactosyltransferase. Catalyzes synthesis of raffinose from galactinol and sucrose. Also involved in degredation. Inferred computationally. 'High confidence'. Paralog of (Glyma.14g010500)
Raffinose Synthase	.	Gm03	Glyma03g29440	Glyma03g29440	Glyma.03g137900	Glyma.03g137900	35393011	35398758	aka galactinol:sucrose 6-galactosyltransferase. Catalyzes synthesis of raffinose from galactinol and sucrose. Also involved in degredation. Inferred computationally. 'High confidence'.
Raffinose Synthase	.	Gm04	Glyma18g23060	Glyma18g23102	Glyma.04g145800	no correspondence	27037731	27039621	aka galactinol:sucrose 6-galactosyltransferase. Catalyzes synthesis of raffinose from galactinol and sucrose. Also involved in degredation. Inferred computationally. 'High confidence'. Aligned to chromosome 18 in v1 assembly.
Raffinose Synthase	.	Gm04	Glyma04g36410	Glyma04g36410	Glyma.04g190000	Glyma.04g190000	46076888	46080907	aka galactinol:sucrose 6-galactosyltransferase. Catalyzes synthesis of raffinose from galactinol and sucrose. Also involved in degredation. Inferred computationally. 'High confidence'.
Raffinose Synthase	*RS3*	Gm05	Glyma05g08950	Glyma05g08950	Glyma.05g003900	Glyma.05g003900	307461	312091	Raffinose Synthase 3. Catalyzes synthesis of raffinose from galactinol and sucrose.
Raffinose Synthase	.	Gm05	Glyma05g02510	Glyma05g02510	Glyma.05g040300	Glyma.05g040300	3593378	3598821	aka galactinol:sucrose 6-galactosyltransferase. Catalyzes synthesis of raffinose from galactinol and sucrose. Inferred computationally. 'High confidence'.

Molecular	Gene symbol	Chromosome	Wm82.a1.v1	Wm82.a1.v1.1	Wm82.a2.v1	Wm82.a4.v1	Start Position (Wm82.a2.v1)	Stop Position (Wm82.a2.v1)	Comments
Raffinose Synthase	.	Gm06	Glyma06g18480	Glyma06g18480	Glyma.06g175500	no correspondence	14845359	14849994	aka galactinol:sucrose 6-galactosyltransferase. Catalyzes synthesis of raffinose from galactinol and sucrose. Also involved in degredation. Inferred computationally. 'High confidence'. Paralog of (Glyma.04g190000)
Raffinose Synthase	*RS2*	Gm06	Glyma06g18890	Glyma06g18890	Glyma.06g179200	Glyma.06g179200	15217419	15223877	Raffinose Synthase 2. Catalyzes synthesis of raffinose from galactinol and sucrose.
Raffinose Synthase	.	Gm09	Glyma09g01940	Glyma09g01940	Glyma.09g016600	Glyma.09g016600	1285133	1290884	aka galactinol:sucrose 6-galactosyltransferase. Catalyzes synthesis of raffinose from galactinol and sucrose. Also involved in degredation. Inferred computationally. 'High confidence'.
Raffinose Synthase	.	Gm13	Glyma13g22890	Glyma13g22890	Glyma.13g160100	Glyma.13g160100	27576192	27579282	aka galactinol:sucrose 6-galactosyltransferase. Catalyzes synthesis of raffinose from galactinol and sucrose. Inferred computationally. 'High confidence'. Paralog of (Glyma.17g111400)
Raffinose Synthase	.	Gm14	Glyma14g01430	Glyma14g01430	Glyma.14g010500	Glyma.14g010500	807239	811633	aka galactinol:sucrose 6-galactosyltransferase. Catalyzes synthesis of raffinose from galactinol and sucrose. Also involved in degredations. Inferred computationally. 'High confidence'.
Raffinose Synthase	.	Gm17	Glyma17g11970	Glyma17g11970	Glyma.17g111400	Glyma.17g111400	8744556	8747526	aka galactinol:sucrose 6-galactosyltransferase. Catalyzes synthesis of raffinose from galactinol and sucrose. Inferred computationally. 'High confidence'.
Raffinose Synthase	.	Gm19	Glyma19g00440	Glyma19g00441	Glyma.19g004400	Glyma.19g004400	359934	363588	aka galactinol:sucrose 6-galactosyltransferase. Catalyzes synthesis of raffinose from galactinol and sucrose. Inferred computationally. 'High confidence'.
Galactinol synthase	.	Gm03	Glyma03g38080	Glyma03g38080	Glyma.03g222000	Glyma.03g222000	42494623	42497111	aka inositol 3-α-galactosyltransferase. Catalyzes the synthesis of galactose. Paralog of (Glyma.19g219100)
Galactinol synthase	.	Gm03	Glyma03g38910	Glyma03g38910	Glyma.03g229800	Glyma.03g229800	43172457	43175687	aka inositol 3-α-galactosyltransferase. Catalyzes the

Molecular	Gene symbol	Chromosome	Wm82.a1.v1	Wm82.a1.v1.1	Wm82.a2.v1	Wm82.a4.v1	Start Position (Wm82.a2.v1)	Stop Position (Wm82.a2.v1)	Comments
									synthesis of galactose. Paralog of (Glyma.19g227800).
Galactinol synthase	.	Gm10	Glyma10g28610	Glyma10g28610	Glyma.10g145300	Glyma.10g145300	38014453	38016396	aka inositol 3-α-galactosyltransferase. Catalyzes the synthesis of galactose. Paralog of (Glyma.20g094500)
Galactinol synthase	.	Gm19	Glyma19g40680	Glyma19g40680	Glyma.19g219100	Glyma.19g219100	47148225	47150373	aka inositol 3-α-galactosyltransferase. Catalyzes the synthesis of galactose.
Galactinol synthase	.	Gm19	Glyma19g41550	Glyma19g41550	Glyma.19g227800	Glyma.19g227800	47911130	47914214	aka inositol 3-α-galactosyltransferase. Catalyzes the synthesis of galactose.
Galactinol synthase	.	Gm20	Glyma20g22700	Glyma20g22700	Glyma.20g094500	Glyma.20g094500	33759417	33761555	aka inositol 3-α-galactosyltransferase. Catalyzes the synthesis of galactose.

Raffinose-Stachyose Degredation Pathway (some enzymes/genes play a role in both synthesis and degradation; duplicate entries are omitted).

Molecular	Gene symbol	Chromosome	Wm82.a1.v1	Wm82.a1.v1.1	Wm82.a2.v1	Wm82.a4.v1	Start Position (Wm82.a2.v1)	Stop Position (Wm82.a2.v1)	Comments
α-Galactosidase	.	Gm14	Glyma19g32250	Glyma19g32250	Glyma.19g140700	Glyma.19g140700	40199041	40201038	Catalyzes the degredation of stachyose to raffinose + α-D-galactose
Galactokinase	.	Gm07	Glyma07g36610	Glyma07g36610	Glyma.07g237700	Glyma.07g237700	41895930	41902181	Catalyzes the degredation of α-D-galactose to 'α-D-galactose 1-phosphate', which ultimately gets converted into UDP-α-D-glucose, which can be used in many other pathways.
Galactokinase	.	Gm08	Glyma08g47350	Glyma08g47350	Glyma.08g358800	no correspondence	47078466	47084413	Catalyzes the degredation of α-D-galactose to 'α-D-galactose 1-phosphate', which ultimately gets converted into UDP-α-D-glucose, which can be used in many other pathways.
Galactokinase	.	Gm17	Glyma17g03990	Glyma17g03990	Glyma.17g035800	Glyma.17g035800	2629012	2639005	Paralog of (Glyma.07g23770). Catalyzes the degredation of α-D-galactose to 'α-D-galactose 1-phosphate', which ultimately gets converted into UDP-α-D-

Molecular	Gene symbol	Chromosome	Wm82.a1.v1	Wm82.a1.v1.1	Wm82.a2.v1	Wm82.a4.v1	Start Position (Wm82.a2.v1)	Stop Position (Wm82.a2.v1)	Comments
									glucose, which can be used in many other pathways.

Sucrose Synthesis / Degradation

Molecular	Gene symbol	Chromosome	Wm82.a1.v1	Wm82.a1.v1.1	Wm82.a2.v1	Wm82.a4.v1	Start Position (Wm82.a2.v1)	Stop Position (Wm82.a2.v1)	Comments
Sucrose synthase	.	Gm02	Glyma02g40740	Glyma02g40740	Glyma.02g240400	Glyma.02g240400	42892680	42898279	aka glycosyltransferase. Catalyzes the degredation of sucrose to β-D-fructofuranose and UDP-α-D-glucose
Sucrose synthase	.	Gm03	no correspondence	Glyma03g37441	Glyma.03g216300	Glyma.03g216300	42037913	42044153	aka glycosyltransferase. Catalyzes the degredation of sucrose to β-D-fructofuranose and UDP-α-D-glucose
Sucrose synthase	.	Gm15	Glyma15g20180	Glyma15g20180	Glyma.15g182600	Glyma.15g182600	17910131	17916426	aka glycosyltransferase. Catalyzes the degredation of sucrose to β-D-fructofuranose and UDP-α-D-glucose
Sucrose synthase	.	Gm11	Glyma11g33240	Glyma11g33240	Glyma.11g212700	Glyma.11g212700	30547238	30552421	aka glycosyltransferase. Catalyzes the degredation of sucrose to β-D-fructofuranose and UDP-α-D-glucose
Sucrose synthase	.	Gm17	no correspondence	Glyma17g05067	Glyma.17g045800	Glyma.17g045800	3404918	3410491	aka glycosyltransferase. Catalyzes the degredation of sucrose to β-D-fructofuranose and UDP-α-D-glucose
Sucrose synthase	.	Gm09	Glyma09g29710	Glyma09g29710	Glyma.09g167000	Glyma.09g167000	39103764	39109664	aka glycosyltransferase. Catalyzes the degredation of sucrose to β-D-fructofuranose and UDP-α-D-glucose
Sucrose synthase	.	Gm09	Glyma09g08550	Glyma09g08550	Glyma.09g073600	Glyma.09g073600	7809852	7816248	Paralog of (Glyma.15g20180). aka glycosyltransferase. Catalyzes the degredation of sucrose to β-D-fructofuranose and UDP-α-D-glucose
Sucrose synthase	.	Gm14	Glyma14g39070	Glyma14g39070	Glyma.14g209900	Glyma.14g209900	47515899	47521687	Paralog of (Glyma.02g240400). aka glycosyltransferase. Catalyzes the degredation of sucrose to β-D-fructofuranose and UDP-α-D-glucose
Sucrose synthase	.	Gm16	Glyma16g34290	Glyma16g34290	Glyma.16g217200	Glyma.16g217200	37414228	37419838	Paralog of (Glyma.09g167000). aka glycosyltransferase. Catalyzes the

Molecular	Gene symbol	Chromosome	Wm82.a1.v1	Wm82.a1.v1.1	Wm82.a2.v1	Wm82.a4.v1	Start Position (Wm82.a2.v1)	Stop Position (Wm82.a2.v1)	Comments
									degredation of sucrose to β-D-fructofuranose and UDP-α-D-glucose
Sucrose synthase	.	Gm19	no correspondence	Glyma19g40041	Glyma.19g212800	Glyma.19g212800	46633685	46639818	Paralog of (Glyma.03g216300). aka glycosyltransferase. Catalyzes the degredation of sucrose to β-D-fructofuranose and UDP-α-D-glucose
Sucrose synthase	.	Gm15	Glyma15g16160	Glyma15g16171	Glyma.15g151000	Glyma.15g151000	12497113	12508050	aka glycosyltransferase. Catalyzes the degredation of sucrose to β-D-fructofuranose and UDP-α-D-glucose
Sucrose synthase	.	Gm13	Glyma13g17420	Glyma13g17421	Glyma.13g114000	Glyma.13g114000	22767704	22773231	Paralog of (Glyma.17g045800). aka glycosyltransferase. Catalyzes the degredation of sucrose to β-D-fructofuranose and UDP-α-D-glucose

Table S3. Soybean gene models putatively involved in flavor characteristics

Molecular	Gene symbol	Chromosome	Wm82.a1.v1	Wm82.a1.v1.1	Wm82.a2.v1	Wm82.a4.v1	Start Position (Wm82.a2.v1)	Stop Position (Wm82.a2.v1)	Comments
Glycitein	*F6H1*	Gm18	Glyma18g08950	Glyma18g08950	Glyma.18g080400	Glyma.18g080400	7708911	7712724	flavone-6-hydroxylase. **NOT expressed in the seed**. Implicated in the synthesis of glycitein, an isoflavone negatively correlated with flavor quality
Glycitein	*F6H2*	Gm18	Glyma18g08930	Glyma18g08930	Glyma.18g080200	Glyma.18g080200	7684214	7686917	flavone-6-hydroxylase. **NOT expressed in the seed**. Implicated in the synthesis of glycitein, an isoflavone negatively correlated with flavor quality
Glycitein	*F6H3*	Gm08	Glyma08g43890	Glyma08g43890	Glyma.08g326900	Glyma.08g326900	44512039	44518672	Paralog of Glyma.18g080200. **EXPRESSED ONLY IN THE SEED**. flavone-6-hydroxylase. Implicated in the synthesis of glycitein, an isoflavone negatively correlated with flavor quality.
Genistein/ Daidzein	.	Gm01	Glyma01g44980	Glyma01g44980	Glyma.01g239300	Glyma.01g239300	56350646	56352389	2-hydroxyisoflavanone dehydratase, catalyzes the formation of genistein and daidzein, two of the three most predominant isoflavonoids in soybean
Genistein/ Daidzein	.	Gm01	Glyma01g44990	Glyma01g44990	Glyma.01g239400	Glyma.01g239400	56356064	56357252	2-hydroxyisoflavanone dehydratase, catalyzes the formation of genistein and daidzein, two of the three most predominant isoflavonoids in soybean
Genistein/ Daidzein	.	Gm01	Glyma01g45000	Glyma01g45000	Glyma.01g239500	Glyma.01g239500	56360162	56361708	2-hydroxyisoflavanone dehydratase, catalyzes the formation of genistein and daidzein, two of the three most predominant isoflavonoids in soybean
Genistein/ Daidzein	.	Gm01	Glyma01g45020	Glyma01g45020	Glyma.01g239600	Glyma.01g239600	56367073	56368854	2-hydroxyisoflavanone dehydratase, catalyzes the formation of genistein and daidzein, two of the three most predominant isoflavonoids in soybean
Genistein/ Daidzein	.	Gm02	Glyma02g15160	Glyma02g15160	Glyma.02g134100	Glyma.02g134100	13865354	13866667	a carboxylesterase in the isoflavonoid biosynthesis pathway
Genistein/ Daidzein	.	Gm07	Glyma07g16660	Glyma07g16660	Glyma.07g138600	no correspondence	16450099	16450096	a carboxylesterase in the isoflavonoid biosynthesis pathway

Molecular	Gene symbol	Chromosome	Wm82.a1.v1	Wm82.a1.v1.1	Wm82.a2.v1	Wm82.a4.v1	Start Position (Wm82.a2.v1)	Stop Position (Wm82.a2.v1)	Comments
Genistein/ Daidzein	.	Gm10	Glyma10g39600	Glyma10g39600	Glyma.10g250200	Glyma.10g250200	47837207	47839071	enzyme with hydrolase activity involved in the isoflavonoid biosynthesis pathway
Genistein/ Daidzein	.	Gm10	Glyma10g39610	Glyma10g39610	Glyma.10g250300	Glyma.10g250300	47841780	47843423	2-hydroxyisoflavanone dehydratase, catalyzes the formation of genistein and daidzein, two of the three most predominant isoflavonoids in soybean
Genistein/ Daidzein	.	Gm11	Glyma11g00650	Glyma11g00650	Glyma.11g004200	Glyma.11g004200	313902	315386	Paralog of Glyma.01g239600. 2-hydroxyisoflavanone dehydratase, catalyzes the formation of genistein and daidzein
Genistein/ Daidzein	.	Gm20	Glyma20g28150	Glyma20g28150	Glyma.20g143400	Glyma.20g143400	38199806	38201866	Paralog of Glyma.10g250200. Enzyme with hydrolase activity involved in the isoflavonoid biosynthesis pathway
Saponins	*Sg-1*	Gm07	Glyma07g38460	Glyma07g38460	Glyma.07g254600	Glyma.07g254600	43138637	43140466	Involved in saponin biosynthesis
Saponins	*Sg-3*	Gm10	Glyma10g16790	Glyma10g16790	Glyma.10g104700	Glyma.10g104700	22965600	22967374	Glucosyltransferase involved in saponin biosynthesis
Saponins	*Sg-4*	Gm01	Glyma01g05500	Glyma01g05500	Glyma.01g046300	Glyma.01g046300	5337434	5338915	Glucosyltransferase involved in saponin biosynthesis
Saponins	*Sg-5*	Gm15	Glyma15g39090	Glyma15g39090	Glyma.15g243300	Glyma.15g243300	46451920	46462166	Involved in saponin biosynthesis
Saponins	.	Gm13	Glyma13g33700	Glyma13g33700	Glyma.13g262100	Glyma.13g262100	36586108	36589827	Paralog of Glyma.15g243300

Table S4. Soybean gene models putatively involved in the tocopherol biosynthesis pathway

Molecular	Gene symbol	Chromosome	Wm82.a1.v1	Wm82.a1.v1.1	Wm82.a2.v1	Wm82.a4.v1	Start Position (Wm82.a2.v1)	Stop Position (Wm82.a2.v1)	Comments
alpha-tocopherol	.	Gm04	Glyma04g08740	Glyma04g08740	Glyma.04g082300	Glyma.04g082300	6945685	6946469	tocopherol cylcase activity (vitamin E biosynthesis), EXPRESSED IN SEED
alpha-tocopherol	.	Gm04	Glyma04g08750	Glyma04g08750	Glyma.04g082500	Glyma.04g082500	6948445	6954177	tocopherol cylcase activity (vitamin E biosynthesis), EXPRESSED IN SEED
alpha-tocopherol	.	Gm04	Glyma04g42270	Glyma04g42270	Glyma.04g243900	Glyma.04g243900	51167017	51173403	tocopherol cylcase activity (vitamin E biosynthesis), NOT heavily expressed in seed
alpha-tocopherol	.	Gm06	Glyma06g08850	Glyma06g08850	Glyma.06g084100	Glyma.06g084100	6466090	6471839	tocopherol cylcase activity (vitamin E biosynthesis) (Paralog of Glyma.04g082500, NOT heavily expressed in seed)
alpha-tocopherol	.	Gm06	Glyma06g12540	Glyma06g12540	Glyma.06g119400	Glyma.06g119400	9719172	9726230	tocopherol cylcase activity (vitamin E biosynthesis), (Paralog of Glyma.04g243900, NOT heavily expressed in seed)

Table S5. Soybean gene models putatively involved in seed oil composition

Fatty Acid Biosynthesis

Molecular	Gene symbol	Chromosome	Wm82.a1.v1	Wm82.a1.v1.1	Wm82.a2.v1	Wm82.a4.v1	Start Position (Wm82.a2.v1)	Stop Position (Wm82.a2.v1)	Comments
ketoacyl-[acp] synthase	*KASI*	Gm08	Glyma08g08910	Glyma08g08910	Glyma.08g084300	Glyma.08g084300	6354366	6359411	Beta-ketoacyl-ACP carrier protein synthase 1 gene. A fast neutron line (FN0176450) with a translocation between chromosome 8 and 13 led to the disruption of this locus, causing increased sucrose and decreased oil.
ketoacyl-[acp] synthase	*KASI*	Gm05	Glyma05g25970	Glyma05g25970	Glyma.05g129600	Glyma.05g129600	32274019	32280372	Paralog of KASI.
ketoacyl-[acp] synthase (fap2)	*KASII-A*	Gm17	Glyma17g05200	Glyma17g05200	Glyma.17g047000	Glyma.17g047000	3524452	3531412	beta-ketoacyl-ACP synthetase 2 gene 1
ketoacyl-[acp] synthase	*KASII-A*	Gm13	Glyma13g17290	Glyma13g17290	Glyma.13g112700	Glyma.13g112700	22631895	22638726	Paralog of KASII-A
ketoacyl-[acp] synthase (fap1)	*KASIII-1*	Gm09	Glyma09g41380	Glyma09g41380	Glyma.09g277400	Glyma.09g277400	49277538	49281994	β-ketoacyl-acyl-carrier-protein synthase III
ketoacyl-[acp] synthase	*KASIII-2*	Gm15	Glyma15g00550	Glyma15g00550	Glyma.15g003100	Glyma.15g003100	276702	278479	β-ketoacyl-acyl-carrier-protein synthase III
ketoacyl-[acp] synthase	*KASIII-3*	Gm18	Glyma18g44350	Glyma18g44350	Glyma.18g211400	Glyma.18g211400	49799310	49803702	β-ketoacyl-acyl-carrier-protein synthase III. Paralog of KASIII-1 (Glyma.09g277400)
SACPD-A	*SACPD, FAB2, SAD1, ACPD*	Gm02	Glyma02g15600	Glyma02g15600	Glyma.02g138100	Glyma.02g138100	14302427	14306293	stearoyl acyl carrier protein, catalyzes the conversion of stearic acid to monounsaturated oleic acid
SACPD-B	*ACPD-2, SAD2*	Gm07	Glyma07g32850	Glyma07g32850	Glyma.07g207200	Glyma.07g207200	37640045	37643952	Paralog of Glyma.02g138100
SACPD-C (fas)	*ACPD-3*	Gm14	Glyma14g27990	Glyma14g27990	Glyma.14g121400	Glyma.14g121400	17499717	17502413	Reduced function in X-ray mutants KK24 and M25 have increased stearic acid. Complete knockout in EMS mutant of Williams82
SACPD	.	Gm13	Glyma13g08990	Glyma13g08986	Glyma.13g038600	Glyma.13g038600	11956631	11959361	Another stearoyl-ACP desaturase, with high sequence similarity to SACPD-C

Molecular	Gene symbol	Chromosome	Wm82.a1.v1	Wm82.a1.v1.1	Wm82.a2.v1	Wm82.a4.v1	Start Position (Wm82.a2.v1)	Stop Position (Wm82.a2.v1)	Comments
FAD (ol)	*FAD2-1A*	Gm10	Glyma10g42470	Glyma10g42470	Glyma.10g278000	Glyma.10g278000	50013484	50015460	Oleate fatty acid desaturase, aka "microsomal omega-6 desaturase".
FAD	*FAD2-1B*	Gm20	Glyma20g24530	Glyma20g24530	Glyma.20g111000	Glyma.20g111000	35315630	35319063	Oleate fatty acid desaturase, aka "omega-6 desaturase"
FAD (fan1)	*FAD3A*	Gm14	Glyma14g37350	Glyma14g37350	Glyma.14g194300	Glyma.14g194300	45935668	45939896	Lineolate fatty acid desaturase, aka "omega-3-fatty acid desaturase 3 gene 1"
FAD (fan3)	*FAD3B*	Gm02	Glyma02g39230	Glyma02g39230	Glyma.02g227200	Glyma.02g227200	41419656	41423881	Lineolate fatty acid desaturase, aka "omega-3-fatty acid desaturase 3 gene 2"
FAD (fan2)	*FAD3C*	Gm18	Glyma18g06950	Glyma18g06950	Glyma.18g062000	Glyma.18g062000	5646502	5649337	Lineolate fatty acid desaturase, aka "omega-3-fatty acid desaturase 3 gene 3"
FAD	*FAD3D*	Gm11	Glyma11g27190	Glyma11g27190	Glyma.11g174100	Glyma.11g174100	19009581	19012951	Lineolate fatty acid desaturase, aka "omega-3-fatty acid desaturase 3 gene 4"
FAD	*FAD7-1*	Gm07	Glyma07g18350	Glyma07g18350	Glyma.07g151300	Glyma.07g151300	18352409	18355476	"chloroplast omega-3 fatty acid desaturase isoform 2"
FAD	*FAD7-2*	Gm18	Glyma18g43210	Glyma18g43210	Glyma.18g202600	Glyma.18g202600	48365045	48368299	"omega-3 fatty acid desaturase 7 gene 2"
FAD	*FAD8-1*	Gm01	Glyma18g15260	Glyma18g15261	Glyma.18g120400	Glyma.18g120400	41337574	41341197	"fatty acid desaturase 8", chloroplast-like
FAD	*FAD8-2*	Gm03	Glyma03g07570	Glyma03g07570	Glyma.03g056700	Glyma.03g056700	7802102	7805630	"omega-3 fatty acid desaturase, chloroplast-like"
FAD	*SLD2-1*	Gm02	Glyma02g11820	Glyma02g11820	Glyma.02g106300	Glyma.02g106300	10139650	10142680	"delta(8)-fatty acid desaturase 2 gene 1"
FAD	.	Gm01	Glyma01g05470	Glyma01g05470	Glyma.01g046000	Glyma.01g046000	5317206	5321170	Paralog of Glyma.02g106300
FAD	*SLD2-2*	Gm08	Glyma08g41120	Glyma08g41120	Glyma.08g299900	Glyma.08g299900	41806629	41809535	"delta(8)-fatty acid desaturase 2 gene 2"
FAD	.	Gm18	Glyma18g15260	Glyma18g15261	Glyma.18g120400	Glyma.18g120400	15086033	15089865	Paralog of Glyma.08g299900
FATB	*FATB1a, fap3-ug*	Gm05	Glyma05g08060	Glyma05g08060	Glyma.05g012300	Glyma.05g012300	1127438	1131632	16:0-acyl carrier protein (ACP) thioesterase enzyme. Mutations in this gene are associated with reduced palmitic acid content. Interestingly, this gene is involved in the conversion of oleoyl-ACP to oleate, which is downstream from the palmitate acid synthesis.
FATB	*FATB1b*	Gm17	Glyma17g12940	Glyma17g12940	Glyma.17g120400	Glyma.17g120400	9570425	9574806	Paralog of Glyma.05g012300. "myristoyl-acyl carrier protein thioesterase".
